# New Immunosuppressive Sphingoid Base and Ceramide Analogues in Wild Cordyceps

**DOI:** 10.1038/srep38641

**Published:** 2016-12-14

**Authors:** Jia-Ning Mi, Yuwei Han, Yingqiong Xu, Junping Kou, Jing-Rong Wang, Zhi-Hong Jiang

**Affiliations:** 1 State Key Laboratory of Quality Research in Chinese Medicine, Macau Institute for Applied Research in Medicine and Health, Macau University of Science and Technology, Macau, China; 2 Jiangsu Key Laboratory of TCM Evaluation and Translational Research, Department of Complex Prescription of TCM, China Pharmaceutical University, 639 Longmian Road, Nanjing 211198, China; 3 International Institute for Translational Chinese Medicine, Guangzhou University of Chinese Medicine, Guangzhou, China

## Abstract

A comprehensive identification of sphingoid bases and ceramides in wild Cordyceps was performed by integrating a sequential chromatographic enrichment procedure and an UHPLC-ultrahigh definition-Q-TOF-MS based sphingolipidomic approach. A total of 43 sphingoid bases and 303 ceramides were identified from wild Cordyceps, including 12 new sphingoid base analogues and 159 new ceramide analogues based on high-resolution MS and MS/MS data, isotope distribution, matching with the comprehensive personal sphingolipid database, confirmation by sphingolipid standards and chromatographic retention time rule. The immunosuppressive bioassay results demonstrated that Cordyceps sphingoid base fraction exhibits more potent immunosuppressive activity than ceramide fraction, elucidating the immunosuppressive ingredients of wild Cordyceps. This study represented the most comprehensive identification of sphingoid bases and ceramides from a natural source. The findings of this study provided an insight into therapeutic application of wild Cordyceps.

Regulating sphingolipid (SPL) metabolism is a promising strategy for immunomodulation^1^,^2^. Natural SPLs represent a classical and well-established source of chemical structure diversity for the development of new chemical entities with immunological effects^3^,^4^. With these premises, global profiling of SPLs in herbal medicines provides a promising platform for the discovery and design of SPL-based immunomodulatory agents.

Cordyceps is a composite consisting of the stroma of the fungus *Cordyceps sinensis* (Berk.) Sacc. (family Hypocreaceae) and the dead caterpillar of *Hepialus armoricanus* (family Hepialidae)^5^. It occupies a prominent position in traditional Chinese medicine owing to its time-honored therapeutic effects for a variety of diseases and broad spectrum of pharmacological activities, e.g., immunomodulatory, anti-inflammatory, antitumor, hypotension and vasorelaxant^6^,^7^. In 1994, myriocin, a natural sphingoid base, was isolated from the culture broth of *Isaria sinclairii* (the imperfect stage of *Cordyceps sinclairii*) as a potent immunosuppressive constituent^8^. FTY720, a derivative of myriocin, was synthesized and developed into the first oral drug (Fingolimod) for the treatment of multiple sclerosis^9^,^10^,^11^. In addition, N-(2′-hydroxy-tetracosanoyl)-2-amino-1,3,4-trihydroxy-octadec-8*E*-ene, a ceramide isolated from Cordyceps, has been reported to have immunomodulatory biological activity^12^. These evidence suggest that sphingoid bases and ceramides should be immunomodulatory active constituents of Cordyceps.

Before the discovery of myriocin, there has been almost no report on SPLs from wild Cordyceps, which might be attributable to the exorbitant price of wild Cordyceps as experimental materials for the isolation and identification of single compounds. Therefore, a well-designed strategy that could facilitate comprehensive identification of the SPLs by using a small amount of herbal materials is of paramount for SPL-based drug discovery.

We herein carried out a comprehensive identification of sphingoid bases and ceramides in wild Cordyceps by integrating a sequential chromatographic enrichment procedure and an improved sphingolipidomic approach established in our lab^13^,^14^. Subsequently, the immunosuppressive activity of fractions of these sphingoid base and ceramide were assayed in lipopolysaccharide (LPS) and concanavalin A (Con A)-induced mouse splenic lymphocyte proliferation models.

## Results

### Analytical strategy for identification of sphingoid bases and ceramides in wild Cordyceps

UHPLC-ultrahigh definition (UHD)-Q-TOF-MS was used to analyze Cordyceps sphingoid base and ceramide fractions which were prepared by using silica gel and amino silica gel column chromatographies (Supplementary Figure S1). Our LC-MS-based sphingolipidomic approach^13^ enabled the elimination of interferences due to isotopic molecules, adduct ions and ion-source collision-induced dissociation; and the discrimination of isobaric and isomeric species. Additionally, a comprehensive personal SPL database was established for reliable screening and identification of sphingoid bases and ceramides. By improving both the detecting method and the SPL database, the present approach can obviously increase the possibility of discovering more sphingoid bases and ceramides.

**Figure 1:**
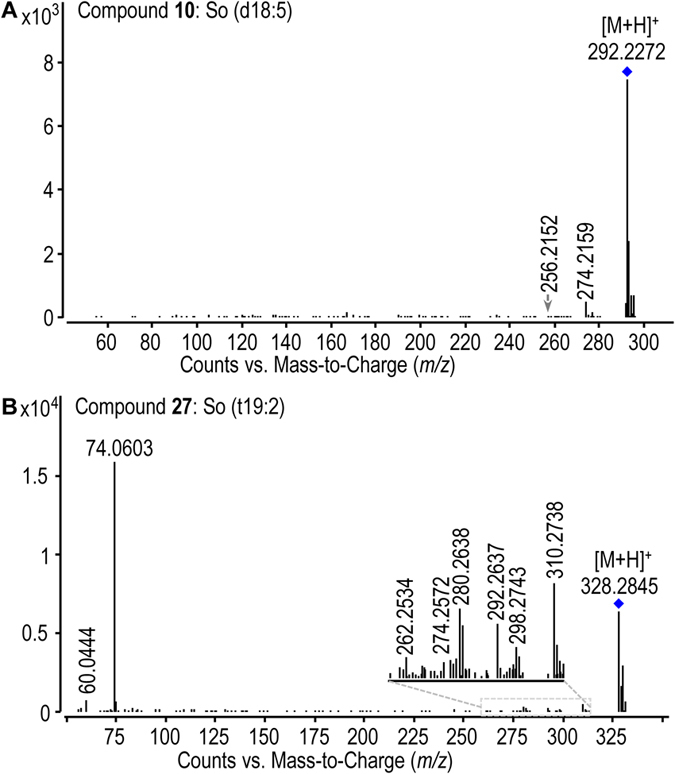
The MS/MS spectra of representative new sphingoid base analogues ((**A**), So (d18:5); (**B**), So (t19:2)).

**Figure 2:**
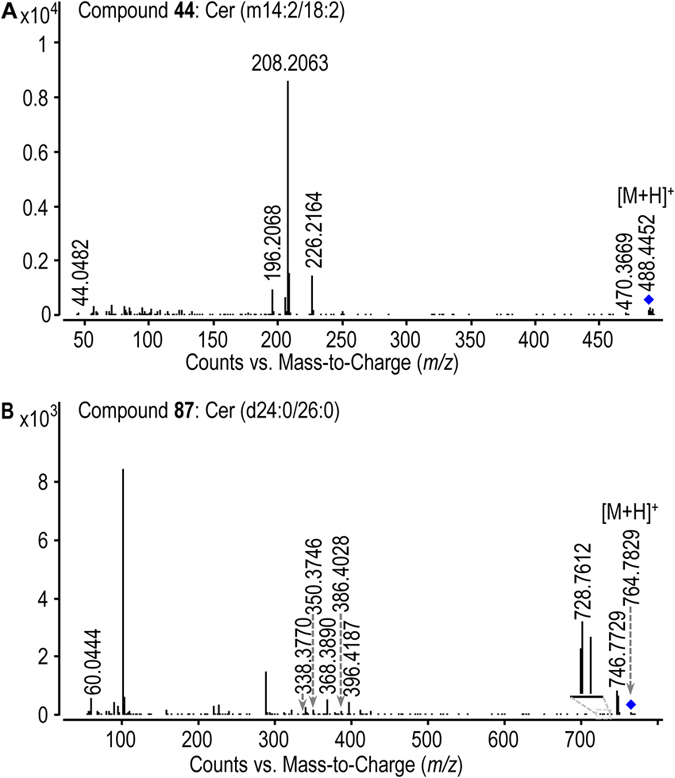
The MS/MS spectra of representative new ceramide analogues ((**A**), Cer (m14:2/18:2); (**B**), Cer (d24:0/26:0)).

**Figure 3:**
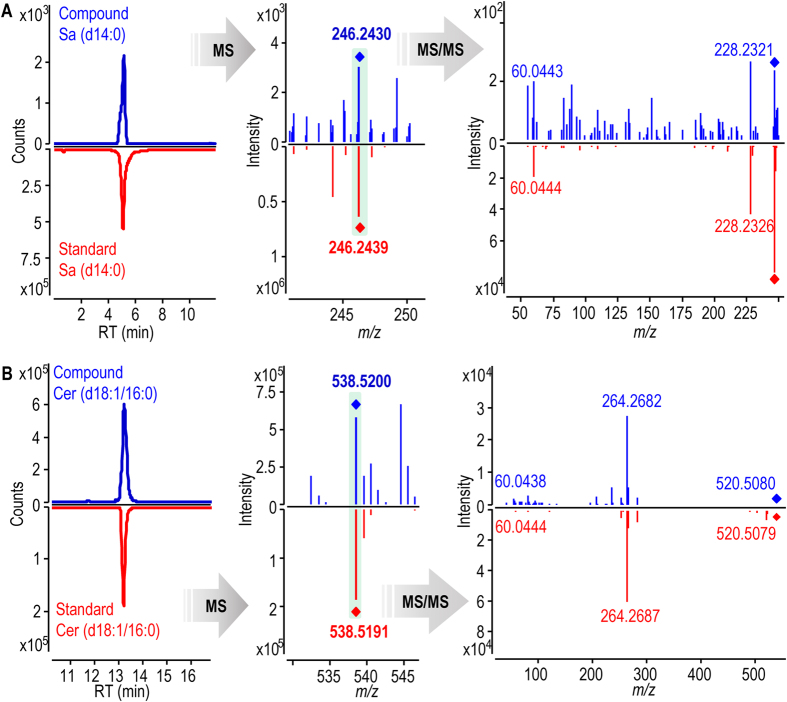
Structural confirmation of Sa (d14:0) (**A**) and Cer (d18:1/16:0) (**B**) in wild Cordyceps by using corresponding standards.

1. Enrichment of sphingoid base and ceramide fractionsSilica gel column chromatography could remove non-SPL components from the total Cordyceps SPL extract greatly, while subsequent NH_2_ silica gel column chromatography could separate SPL species by class efficiently, thus facilitating successful fractionation of sphingoid bases and ceramides. By using this sequential column chromatography procedure, ion-suppression arising from non-SPL impurities and isobaric/isotopic interference derived from co-eluted SPL species can be greatly reduced, resulting in significantly improved signal intensities and mass accuracy of low-abundance SPL species in the MS detection. Additionally, the reduced isobaric/isotopic interference ensured high quality MS/MS detection of target SPL species. The greatly improved MS and MS/MS detection laid firm basis for the reliable identification of these low-abundance SPL species that can not be identified by using routine approach. A significant increase in the MS signal response of low-content and/or low-abundance SPL species in the LC-MS analyzes of Cordyceps via the enrichment of sphingoid bases and ceramides was achieved. For example, signal response of some low-abundance sphingoid bases (Supplementary Figure S2, sphingoid bases **4**, **10**, **22** and **31**) in LC-MS were enhanced after the enrichment procedure. Additionally, lots of low-content ceramides have been discovered in ceramide-enriched fraction. This can be exemplified by dihydroceramides, e.g., d14:0 dihydroceramides (ceramides **63**–**66**) and long-chain dihydroceramides [Cer (d18:0/25:0) (**81**), Cer (d18:0/26:0) (**82**), Cer (d20:0/25:0) (**83**), Cer (d22:0/25:0) (**85**)]; ceramides with long-N-acyl-chain, e.g., C28 ceramide (**236**) and C32 ceramides (**345**); polyhydroxyl ceramides, e.g., Cer (t14:1/25:5(tOH)) (**299**); and polyunsaturated ceramides, e.g., Cer (t14:1/25:5(tOH)) (**299**) and Cer (d18:2/16:3) (**317**). All evidence indicated the importance of SPLs separation strategy for the deep profiling of sphingoid bases and ceramides in Cordyceps.
2. Structural elucidation of sphingoid bases and ceramides in wild CordycepsThe structures of sphingoid bases and ceramides were elucidated by means of UHPLC-UHD-Q-TOF-MS/MS. The feature ions specific to the sphingoid backbone and the fatty acid chain are decisive for the identification of sphingoid bases and ceramides, e.g., *m/z* 210 and *m/z* 364 ions for the assignation of C14 and C25 sphingoid backbones, respectively^15^; *m/z* 400 and *m/z* 358 ions (diagnostic ions for C24 fatty acid chain with five double bonds) for the discovery of ceramides with polyunsaturated fatty acid chain. Additionally, the neutral loss information of H_2_O and/or HCHO aids in the identification of polyhydroxyl sphingoid bases and ceramides. The configuration of some asymmetric carbons in SPLs were assigned based on the *de novo* SPL biosynthetic pathway reported in the reference (Supplementary Figure S3). As a fungi, possible SPLs synthesis process of Cordyceps may involve a *de novo* SPL biosynthetic pathway in all eukaryotic cells^16^,^17^,^18^, metabolism of fungi phytosphingolipid^19^,^20^, as well as biosynthetic pathway of non-traditional 1-deoxysphingoid bases^21^,^22^. These publications reported common sphingolipids metabolism existing in all eukaryotic cells and special biosynthetic pathway of sphingolipids in fungi, which provided important evidence to describe the plausible biosynthetic pathway of sphingolipids in Cordyceps. The configuration of CH-N (C2) asymmetric carbon was assigned as *S* because this carbon is primitively derived from L-serine (step 1). The configuration of CH-O asymmetric carbon (C3) was confirmed as *R* form according to the step that generates sphinganine [(2 *S*, 3 *R*)-2-aminooctadecane-1, 3-diol] (step 2). Since sphinganine is converted into phytosphingosine [(2 *S*, 3 *R*, 4 *R*)-2-aminooctadecane-1, 3, 4-triol] by C4 hydroxylase in step 4, *R* configuration of the CH-O asymmetric carbons in phytosphingoid bases (C4) and associated ceramides was demonstrated.Sphingoid bases were identified based on accurate mass of the protonated precursor ion ([M + H]^+^) and the protonated product ion information by loss of a H_2_O ([M + H-H_2_O]^+^). For instance, compound **10** with the protonated precursor ion at *m/z* 292.2272 was matched to be So (d18:5) by using the SPL database based on its accurate mass. In its MS/MS spectrum (Fig. 1A), the information of ions at *m/z* 274 ([M + H-H_2_O]^+^) and *m/z* 256 ([M + H-2H_2_O]^+^) confirmed its structure.Additionally, the neutral loss information of H_2_O and/or HCHO observed from MS/MS spectrum, is very valuable to identify the number of hydroxyl group in sphingoid bases. This can be exemplified by the characterization of So (t19:2) (**27**), whose [M + H]^+^ ion at *m/z* 328.2845 matched with a C19 sphingosine with three hydroxyl groups and two double bonds by using the SPL database based on its accurate mass. In its MS/MS spectrum (Fig. 1B), the protonated precursor ion at *m/z* 328 yielded three feature ions at *m/z* 310 ([M + H-H_2_O]^+^), *m/z* 292 ([M + H-2H_2_O]^+^) and *m/z* 274 ([M + H-3H_2_O]^+^) by loss of H_2_O, indicating that three hydroxyl groups exist in this sphingosine. The identification of So (t19:2) was also supported by ions at *m/z* 298 ([M + H-HCHO]^+^), *m/z* 280 ([M + H-HCHO-H_2_O]^+^) and *m/z* 262 ([M + H-HCHO-2H_2_O]^+^) produced from the protonated precursor ion by loss of HCHO and H_2_O.The structural elucidation of ceramides mainly was based on the feature ion representative of the sphingoid backbone, because the characteristic fragment reflecting the fatty acid chain was low-abundance, especially for the minor ceramides. For example, compound **44** with the protonated precursor ion at *m/z* 488.4452 was assigned as a 1-deoxyl ceramide with four double bonds by matching with the SPL database. The fragment clues in its MS/MS spectrum were used for the structural elucidation (Fig. 2A): (1) The ion at *m/z* 470 was yielded by loss of a H_2_O, which suggested that this ceramide has one hydroxyl group; (2) A low-abundance ion of *m/z* 226 was produced by the elimination of the fatty acid chain from the protonated precursor ion, which is a protonated C14 deoxysphingosine with two double bonds; (3) The ion species of *m/z* 208 and *m/z* 196 arising from the *m/z* 226 ion via loss of H_2_O and HCHO, respectively, supported that one hydroxyl group exists in sphingoid backbone of this ceramide; (4) A feature ion at *m/z* 44 of 1-deoxyl sphingoid backbone ceramide was observed. All evidence indicated that this ceramide should be Cer (m14:2/18:2) (**44**). Another example, based on the accurate mass of the protonated precursor ion (*m/z* 764.7829), compound **87** was decided as a ceramide with the formula C_50_H_101_NO_3_ by matching with the SPL database. Its MS/MS spectrum provided important fragment information for the structural elucidation (Fig. 2B), e.g., the protonated precursor ion yielded the *m/z* 746 ion and *m/z* 728 ion by loss of H_2_O, indicating that this ceramide contains two hydroxyl groups; the direct cleavage of the C2-N bond gave rise to the *m/z* 396 ion, which reflects a C26 fatty acid chain; the elimination of the fatty acid chain from the protonated precursor ion resulted in a low-abundance ion at *m/z* 386 which is a protonated C24 sphinganine; the *m/z* 386 ion yielded the *m/z* 368 ion which yielded the ion species of *m/z* 350 and *m/z* 338 via loss of H_2_O and HCHO, respectively, which further confirmed d24:0 sphingoid backbone. Based on these fragment clues mentioned above, this ceramide was identified as Cer (d24:0/26:0) (**87**).
3. Confirmation of sphingolipids by using commercial and synthesized standardsIn order to confirm the structures of sphingoid bases and ceramides, 19 commercial SPL standards (Supplementary Table S1, sphingoid bases **2**, **9**, **23**, **37** and **38**; ceramides **70**, **72**, **73**, **79**, **80**, **111**, **144**, **178**, **182**, **185**, **188**, **194**, **195** and **239**) and 3 authentic SPLs synthesized by us (Supplementary Figure S4, sphinganine **1**, ceramides **75** and **77**) were used as markers in UHPLC-MS analysis of wild Cordyceps. Firstly, the comparison of chromatographic retention time (RT) showed that 6 sphingoid bases and 16 ceramides in wild Cordyceps have same RT values with corresponding SPL standards (Supplementary Figure S5). Secondly, the structures of 22 SPLs in wild Cordyceps were further confirmed by comparing their high-resolution MS and MS/MS spectra with those of corresponding SPL standards. For instance, Fig. 3A displayed that the structure of compound **1** in wild Cordyceps was confirmed by using Sa (d14:0) standard based on the same chromatographic RT at 4.99 min and accurate masses of protonated precursor ion at *m/z* 246 and characterized fragment ion at *m/z* 228 ([M-H_2_O + H]^+^). Another example, the structure of compound **178** in wild Cordyceps was confirmed by using Cer (d18:1/16:0) standard, which was implemented by comparing RT, high-resolution MS, and MS/MS pattern of this ceramide with those of the standard (Fig. 3B).
4. Applications of the retention time in identification of ceramides

Based on the identification of ceramides, linear regression models were constructed by plotting carbon number versus (vs.) RT of ceramides sharing the same sphingoid backbone and unsaturated degree (Supplementary Figure S6). Goodness of fit (R^2^ > 0.998) implied its capability for predicting chromatographic retention of some ceramides, as well as for aiding in identification. In this work, the chromatographic RT rule was used for the identification of low-abundance ceramide species without enough diagnostic fragment information. For example, in the MS/MS data of Cer (d18:1/26:1) (Supplementary Table S1, ceramide **203**), the loss of a H_2_O obtained from *m/z* 282 ion to *m/z* 264 ion only indicates that at least one of hydroxyl group exists in the sphingoid backbone. Due to lacking of feature ions in its MS/MS data reflecting the fatty acid chain, ceramide **203** was identified as either Cer (m18:2/26:0(OH)) or Cer (d18:1/26:1). The chromatographic RT rule was applied to make sure its structure as Cer (d18:1/26:1) (**203**), which was implemented by the investigation that the RT at 19.23 min of ceramide **203** is suitable to the linear regression model of Cer (d18:1/x:1) with R^2 ^= 0.9987 (Supplementary Figure S6). This strategy was also used to support the identification of 9 polyunsaturated ceramides, such as Cer (d18:1/32:4), Cer (d18:2/18:2), Cer (d18:2/18:3), Cer (d18:2/20:4), Cer (d18:2/22:3), Cer (d18:2/23:3), Cer (d18:2/23:5), Cer (d18:2/24:3) and Cer (d18:2/25:5) (Supplementary Table S1, ceramides **210**, **318**, **319**, **321**, **325**, **328**, **329**, **333** and **336**).

### Chemical characteristics of new analogues of sphingoid base and ceramide in wild Cordyceps

Based on the rigorous structural elucidation of sphingoid bases and ceramides mentioned above, these newly characterized sphingoid bases and ceramides significantly enlarged structural diversification of natural SPLs, which were described class by class as below.

**Figure 4:**
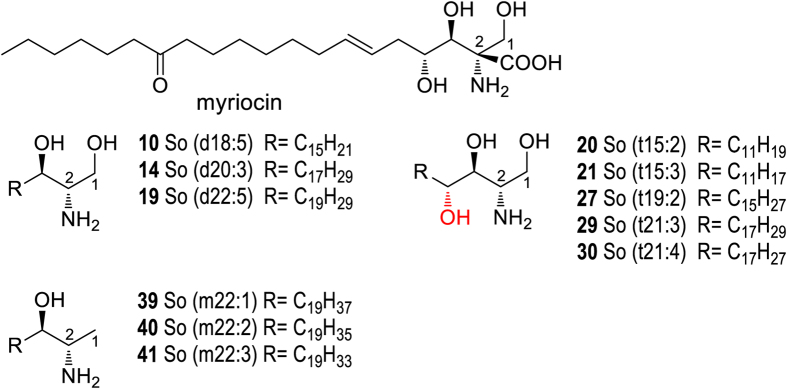
Structures of myriocin and new sphingoid base analogues.

1. New sphingoid base analoguesThe newly characterized sphingoid bases in Cordyceps represented three novel structural features (Fig. 4). The first one is the high unsaturation degree (3 to 5) on C18-C22 alkyl chain, as shown by compounds **10**, **14** and **19**. The second one is the multiple unsaturation degree occurs on odd-numbered sphingoid bases, as evidenced by compounds **20**, **21**, **27**, **29** and **30**. The third feature is the as long as 22 carbon chain length of 1-deoxysphingosines (**39**–**41**). Since the maximum carbon chain length of the previously reported 1-deoxysphingosines is 20^23^, our result provided the first example of the extended carbon chain length for this group of structures. Upon the comprehensive identification, the structural variations of sphingoid bases in Cordyceps can be summarized as: (1) Chain length varied from C14 to C23; (2) Degree of unsaturation from 0 to 5; and (3) The number of hydroxyls varied from 1 to 3.
2. New ceramide analogues

**Figure 5:**
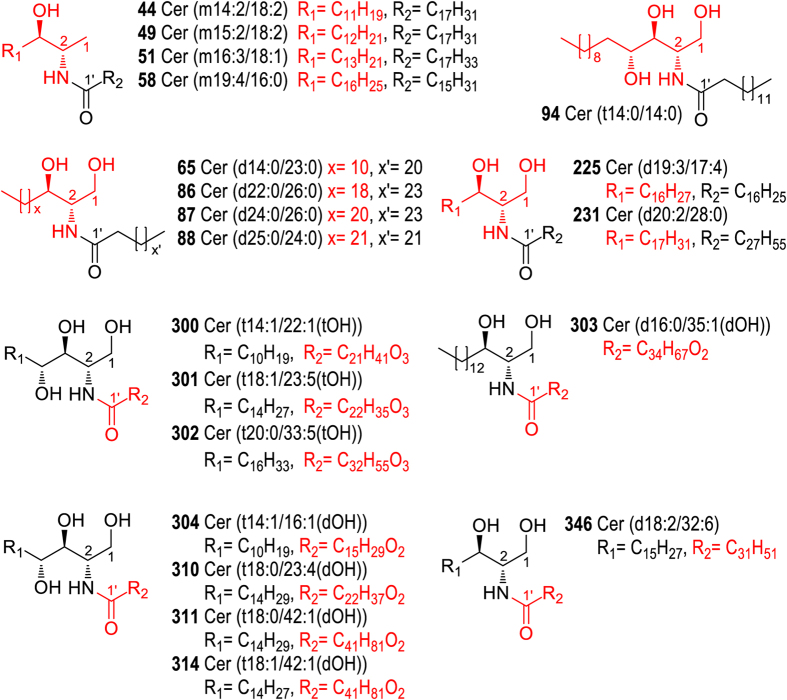
Structures of representative new ceramide analogues.

Ceramides are the most structurally diversified species in Cordyceps. A total of 303 ceramides were characterized from Cordyceps, among which 159 were new ceramide analogues. Ceramides are formed via acylation of sphingoid backbone by long-chain fatty acids. Therefore, structural diversity of ceramides can be derived from either sphingoid backbone or fatty acids^21^. In Cordyceps, a large number of new ceramide analogues are derived from sphingoid backbones that were not previously reported in ceramides (Fig. 5).

The sphingoid bases in the new ceramides include: (1) C14, C15, C16 and C19 1-deoxysphingosine (**44**, **49**, **51** and **58**), which represent the first example of such backbones with unusual chain length^24^; (2) Sphingoid backbones with very short chain (C14) and long chain (C22, C24 and C25) (**65**, **86**–**88**, **94**); (3) Polyunsaturated sphingoid backbones, e.g., d19:3 and d20:2 ceramides (**225**, **231**).

New ceramide analogues arising from the alteration of fatty acids includes: (1) Ceramides with polyhydroxyl fatty acid chains, as exemplified by ceramides **300**–**302** whose fatty acid chains are tri-hydroxylated^25^; Similarly, dihydroxylated fatty acids linked to rare d16:0 and t14:1 sphingoid backbones instead of known t18:1 and t18:0 sphingoid backbone^26^ were identified for the first time (ceramides **303** and **304**); (2) Ceramides with extremely-long fatty acid chains, as represented by Cer (t18:0/42:1(dOH)) (**311**), Cer (t18:1/42:1(dOH)) (**314**) and Cer (d16:0/35:1(dOH)) (**303**); (3) Ceramides with polyunsaturated fatty acids (with unsaturation degree of 4–6), as exemplified by compounds **310**, **346**, suggesting a largely increased unsaturation degree of fatty acid chain^27^.

### Immunosuppressive activities of fractions of Cordyceps sphingoid base and ceramide

Assay of immunosuppressive activity showed that sphingoid base and ceramide fractions of Cordyceps could inhibit LPS and Con A-induced proliferation of mouse splenic lymphocyte^28^ in a dose-dependent manner (Supplementary Figure S7). In LPS-induced mouse splenic lymphocyte proliferation assay, the IC_50_ values of sphingoid base and ceramide fractions were determined to be 3.61 μg/mL and 6.09 μg/mL, respectively, showing that Cordyceps sphingoid base fraction exhibits more potent immunosuppressive activity than ceramide fraction; In Con A-induced mouse splenic lymphocyte proliferation assay, their IC_50_ values were determined to be 1.85 μg/mL and 1.86 μg/mL, respectively, further confirmed the potent immunosuppressive activity of sphingoid base and ceramide fractions of Cordyceps.

## Discussion

The major goal of this study was to comprehensively profile sphingoid bases and ceramides in wild Cordyceps, and to determine the immunosuppressive activity of these SPL fractions, so as to provide evidence for its rational development and clinical application.

No matter how the ordinary extraction method is specific and selective, the crude SPL extract often contains various subclasses of SPLs and non-SPL constituents. The non-SPL species and high-abundance SPL species (e.g., sphingomyelins) may result in significant influence on the detection of low-abundance SPLs (e.g., sphingoid bases, dihydroceramides and polyunsaturated ceramides) in MS, such as ionization suppression and isobaric interference^13^. Therefore, enrichment is a feasible approach for enhancing the detection of SPLs as this procedure can reduce ionization suppression and isobaric interference efficiently.

In the current study, we described a method for the enrichment of sphingoid bases and ceramides from the total SPLs extract by using silica gel and amino silica gel column chromatographies. Silica gel column chromatography could remove non-SPL components from the total Cordyceps SPL extract greatly, while subsequent NH_2_ silica gel column chromatography could separate SPL species by class efficiently. Employment of this enrichment procedure led to greatly reduce ion-suppression arising from non-SPL impurities and isobaric/isotopic interference derived from co-eluted SPL species, resulting in significantly enhanced MS and MS/MS signal response of low-content and/or low-abundance SPL species, such as polyunsaturated sphingosine and dihydroceramides. The enhanced signal response in turn resulted in almost 6-fold increase in the number of identified sphingoid bases and ceramides. Notably, a considerable number of new SPL analogues (12 sphingoid bases and 159 ceramides) were successfully identified by using this approach. The number of new analogues accounted for nearly 50% of the total number of identified SPLs in Cordyceps, showing significant improvement in the SPLs profiling. In summary, this study represents the most comprehensive identification of sphingoid bases and ceramides from a natural source, suggesting great potential of Cordyceps as a resources for the exploration of natural SPLs.

Our study not only indicated structural diversity of SPLs in wild Cordyceps, but also revealed specific SPLs in Cordyceps. Compared to the SPLs in animals^13^,^21^, Cordyceps has been found to contain a series of uncommon sphingoid bases, i.e., shorter chains (e.g., C14, C15) and longer chains (e.g., C22), 4-hydroxyed, and 1-dehydroxyed, as well as ceramides with uncommon sphingoid backbones, e.g., shorter chains, longer chains, 4-hydroxyed, and 1-dehydroxyed and/or uncommon N-acyl chains such as longer N-acyl chains (e.g., C27-C42), and polyhydroxylated etc.

Current study further showed the potent immunosuppressive activities of fractions of sphingoid base (0.08% of crude drug) and ceramide (0.11% of crude drug) of Cordyceps, indicating that we have largely clarified the active SPLs responsible for the immunosuppressive effects of Cordyceps at subclass level. Previous studies on the structure-activity relationship of myriocin, a sphingoid base isolated from *Isaria sinclairii* (the imperfect stage of *Cordyceps sinclairii*), have shown that 2-substituted 2-aminoethanol is the minimum essential structure for the immunosuppressive activity of myriocin^29^. Selective acetylation or complete acetylation of the amino group greatly decreased the immunosuppressive activity of myriocin^30^,^31^. More recently, it was revealed that sphingoid base analogs are substrates of sphingosine kinase (SPHKs) due to their structural homology to sphingosine. The resultant 1-phosphate of sphingosine base analogs acts through sphingosine 1-phosphate signaling pathways to exert immunosuppressive activity^32^,^33^,^34^. This might be the mechanism responsible for more potent immunosuppressive activity of sphingoid bases as compared to ceramides. Because Cordyceps sphingoid base fraction exhibits more potent immunosuppressive activity than ceramide fraction, the immunosuppressive activity of SPLs in Cordyceps might be subclass-dependent. Although the IC_50_ values of fractions of sphingoid base and ceramide are higher about 20-fold and 40-fold (about 17-fold in Con A-induced mouse splenic lymphocyte proliferation assay) than that of FTY720 (positive control), respectively, in SPL-induced mouse splenic lymphocyte proliferation assay, wild Cordyceps holds great promise as a natural resource for the discovery of single SPL with potent immunosuppressive activity.

Due to the intrinsic complexity of SPLs in Cordyceps, as well as the extremely high value of wild Cordyceps, it’s infeasible to isolate individual SPL for further bioassay. Therefore, we assayed the immunosuppressive activities of sphingoid base and ceramide fractions, and demonstrated their potent immunosuppressive activity for the first time. With the information of fraction yield, we could largely clarify the active SPLs responsible for the immunosuppressive effects of Cordyceps at subclass level. At the same time, we profiled SPLs in each fraction comprehensively, revealing detailed structure variations of each subclass. The results provided valuable guidance for preparing representative SPLs for bioactivity assay, which could facilitate identification of individual SPLs with immunosuppressive activity eventually.

## Materials and Methods

### Chemicals

Methanol (MeOH, LC-MS grade), isopropanol (IPA, LC-MS grade), chloroform (CHCl_3_, HPLC grade), acetone (HPLC grade), hexane (HPLC grade), ethyl acetate (EtOAc, HPLC grade) were purchased from Avantor Performance Materials, Inc. (Center Valley, PA, USA). Formic acid (LC-MS grade), acetic acid (LC-MS grade), ammonium acetate (purity ≥ 98%), potassium hydroxide (KOH, purity ≥ 85%) and dimethyl sulfoxide (DMSO, purity ≥ 99%) were purchased from Sigma-Aldrich (St. Louis, MO, USA). Distilled water was prepared using a Milli-Q system (Millipore, Billerica, MA). Davisil^®^ silica media (GRACE 710 NW, Particle size 10–14 μm) and Davisil^®^ amino silica media (GRACE 633 NNH2, Particle size 35–70 μm) were purchased from Grace (Columbia, MD, USA). Lipopolysaccharide and concanavalin A were purchased from Sigma-Aldrich (St. Louis, MO, USA). RPMI 1640 was purchased from Gibco (Invitrogen, Carlsbad, NM, USA). 3-(4, 5-dimethylthiazol-2-yl)-2, 5-diphenyltetrazolium bromide (MTT) was purchased from Amersco LLC. (Cochran Road Solon, OH, USA). Fetal Bovine Serum (FBS) was purchased from Zhejiang Tian Hang Biological Technology Stock Co., Ltd. (Zhejiang, China). SPL standards So (m18:1), Sa (m18:0), So (d18:1), Sa (t18:0), Cer (d18:1/16:0), Cer (d18:0/16:0), Cer (18:1/18:0), Cer (18:0/18:1), Cer (18:0/18:0), Cer (18:1/20:0), Cer (18:1/22:0), Cer (18:1/24:1), Cer (18:1/24:0), Cer (18:0/24:1), Cer (d18:0/24:0), Cer (d18:0/24:0(OH)), Cer (t18:0/24:0), Cer (t18:0/24:0(OH)) were obtained from Avanti Polar Lipids (AL, USA). So (d14:1) was obtained from Matreya LLC (PA, USA). FTY720 (purity > 98%) was purchased from Santa Cruz Biotechnology, Inc. (Texas, USA). Three standards Sa (d14:0), Cer (18:0/20:0) and Cer (d18:0/22:0) were synthesized by reduction of the backbone double bond using hydrogen gas and 10% Pd on charcoal (Aldrich-Sigma, St. Louis, MO), and the conversion was verified by UHPLC-UHD-Q-TOF MS/MS. Nomenclature of SPL species is conducted according to LIPID MAPS (Lipidomics Gateway) nomenclature system. For ceramides, annotation of sphingoid backbone denotes number of hydroxyl group, number of carbon and number of unsaturation degree (e.g., m means one hydroxyl group; d means two hydroxyl groups; t means three hydroxyl groups); annotation of N-acyl chain indicates number of carbon, number of unsaturation degree and number of hydroxyl group (e.g., in d18:1/24:0(OH), 24 means number of total carbon; 0 means number of unsaturation degree; OH means one hydroxyl group). For some ceramides with polyhydroxyl N-acyl chain, dOH and tOH mean two hydroxyl groups and three hydroxyl groups in N-acyl chain, respectively. For example, Cer (t18:1/42:1(dOH)) and Cer (t14:1/22:1(tOH)).

### Animals

Male ICR mice (Weight, 20 to 22 g) were provided by the Comparative Medicine Center of Yangzhou University, China. The animal studies were approved by the Animal Ethics Committee of China Pharmaceutical University. All methods for the study on animals were carried out in accordance with the approved guidelines.

### Preparation of sphingoid base and ceramide fractions

Total SPL extract was prepared following the procedures developed in our previous studies^13^,^14^. Briefly, 65 g of powdered wild Cordyceps was accurately weighed into a glass bottle, in which 1 L of CHCl_3_/MeOH (1:2, v/v) was added. The mixture was incubated at 48 °C for 12 h. After filtration, the remaining residue was ultrasound-assisted extracted with 1 L of CHCl_3_/MeOH (1:2, v/v) for 30 min. The filtrate was collected and the remaining residue was ultrasound-assisted extracted twice with 1 L of CHCl_3_/MeOH (2:1, v/v) for 30 min each time. All filtrates were combined and concentrated to 400 mL by using a rotary evaporator. Then, 40 mL of KOH in MeOH (1 M) was added and the mixture was incubated at 37 °C with shaking for 2 h. The resultant extract was then neutralized with 0.7 mL of acetic acid and centrifuged. The supernatant was evaporated to afford the crude SPLs which was fractionated by silica gel column chromatography (4.2 cm I.D. × 29 cm) with 5 bed volume (BV) of CHCl_3_, 3.5 BV of acetone/MeOH (9:1, v/v) and finally with 7.5 BV of MeOH to give enriched sphingoid base and ceramide fractions. Then, these two fractions were further purified respectively to give sphingoid base fraction (49.6 mg) and ceramide fraction (68.1 mg) by NH_2_ silica gel column chromatography (4.2 cm I.D. × 25 cm) with 2 BV of hexane, 6 BV of hexane/EtOAc (85:15, v/v), 5 BV of CHCl_3_/MeOH (23:1, v/v), 5 BV of acetone/MeOH (9:1.35, v/v) and 5 BV of CHCl_3_/MeOH (2:1, v/v). The sphingoid base and ceramide fractions were dried and reconstituted in methanol. All solutions were filtered through a 0.22 μm filter before LC-MS analysis.

### LC-MS conditions

For detection of sphingoid bases and ceramides, an optimized UHPLC-UHD-Q-TOF MS approach in our lab^13^,^14^ was used in this work. For chromatographic separation, an Agilent 1290 Infinity UHPLC system (Santa Clara, CA, USA) equipped with a binary solvent delivery system, a standard autosampler, and an Agilent Eclipse Plus C18 column (100 × 2.1 mm, 1.8 μm) was used. MeOH/H_2_O/formic acid (60:40:0.2, v/v/v) (A) and MeOH/IPA/formic acid (60:40:0.2, v/v/v) (B), both of which contained 10 mM ammonium acetate were used as eluents at a flow rate of 0.35 mL/min. MS and MS/MS analyses were carried out on an Agilent UHD 6550 Q-TOF mass spectrometer (Santa Clara, CA, USA) with a Jet Stream electrospray ionization source in the positive ion mode. Mass spectra were recorded across the range of *m/z* 200–1700; MS/MS spectra were recorded across the range of *m/z* 40–1700. Targeted MS/MS collision energies were from 10 to 60 eV.

### Immunosuppressive activity assay

ICR mice were killed by cervical dislocation, and the spleens were removed aseptically. Mononuclear cell suspensions were prepared and re-suspended in RPMI 1640 medium (containing 10% FBS). The resulting splenic lymphocytes suspensions (4 × 10^6^ cells/mL) were cultured in 96-well plate (100 μL of suspensions each well). In control group, 100 μL of 10% FBS in RPMI1640 was added into each well (five replicates of wells). In LPS or Con A model group, 100 μL of LPS or Con A (2.5 mg/L of final concentration) was added into each well (five replicates of wells). For the positive drug treatment group, 50 μL of LPS or Con A (2.5 mg/L of final concentration) and 50 μL of FTY720 with a set of final concentrations (0.001, 0.003, 0.01, 0.03, 0.1, 0.3, 1, 3 and 10 μM) were added into each well with five replicates of wells. In test group, 50 μL of LPS or Con A (2.5 mg/L of final concentration) and 50 μL of sphingoid base or ceramide fractions with a set of final concentrations were added into each well with five replicates of wells. The final concentrations of sphingoid base and ceramide fractions were: 0.01, 0.03, 0.1, 0.3, 1, 3, 10, 30 and 100 μg/mL. All splenic lymphocytes in different groups were cultured for 48 h. After the culture, 20 μL of MTT was pulsed each well for 4 h of incubation. After that, the mixture was centrifuged and the supernatant was removed. 150 μL of DMSO was added into each well for 10 min shaking. Then the OD_490_ readings were taken with a microplate reader (Bio Tek, Tigan Street Winooski, VT, USA).

### Data processing and statistical analysis

The inhibitory concentration of the compound that reduced cell proliferation by 50% (IC_50_) values were determined by using the GraphPad Prism 5 software. Student’s t-test or Dunnett’s test was used to analyze data and compare groups.

## Additional Information

**How to cite this article:** Mi, J.-N. *et al*. New Immunosuppressive Sphingoid Base and Ceramide Analogues in Wild Cordyceps. *Sci. Rep.* **6**, 38641; doi: 10.1038/srep38641 (2016).

**Publisher's note:** Springer Nature remains neutral with regard to jurisdictional claims in published maps and institutional affiliations.

## Supplementary Material

Supplementary Information

## References

[b1] Bondy G. S. & Pestka J. J. Immunomodulation by fungal toxins. J. Toxicol. Environ. Health B Crit. Rev. 3, 109–143 (2000). 10834078 10.1080/109374000281113

[b2] Diesner S. C. . Perspectives on immunomodulation early in life. Pediatr. Allergy Immunol. 23, 210–223 (2012). 22299601 10.1111/j.1399-3038.2011.01259.x

[b3] Beckham T. H., Cheng J. C., Marrison S. T., Norris J. S. & Liu X. Interdiction of sphingolipid metabolism to improve standard cancer therapies. Adv. Cancer Res. 117, 1–36 (2013). 23290775 10.1016/B978-0-12-394274-6.00001-7 PMC4203652

[b4] Delgado A., Fabriàs G. Casas J. & Abad J. L. Natural products as platforms for the design of sphingolipid-related anticancer agents. Adv. Cancer Res. 117, 237–281 (2013). 23290782 10.1016/B978-0-12-394274-6.00008-X

[b5] The Pharmacopoeia Commission of PRC (Eds), Pharmacopoeia of the People’s Republic of China, Chemical Industry Publishing House, Beijing, pp. 36 (2010).

[b6] Lo H. C., Hsieh C., Lin F. Y. & Hsu T. H. A Systematic Review of the Mysterious Caterpillar Fungus Ophiocordyceps sinensis in Dong-ChongXiaCao (Dong Chong Xia Cao) and Related Bioactive Ingredients. J. Tradit. Complement Med. 3, 16–32 (2013). 24716152 10.4103/2225-4110.106538 PMC3924981

[b7] Zhou X., Gong Z., Su Y., Lin J. & Tang K. Cordyceps fungi: natural products, pharmacological functions and developmental products. J. Pharm. Pharmacol. 61, 279–291 (2009). 19222900 10.1211/jpp/61.03.0002

[b8] Fujita T. . Fungal metabolites. Part 11. A potent immunosuppressive activity found in *Isaria sinclairii* metabolite. J. Antibiot. 47, 208–215 (1994). 8150717 10.7164/antibiotics.47.208

[b9] Adachi K. . Design, synthesis, and structure-activity relationships of 2-substituted-2-amino-1,3-propanediols: Discovery of a novel immunosuppressant, FTY720. Bioorg. Med. Chem. Lett. 5, 853–856 (1995).

[b10] Kappos L. . Oral fingolimod (FTY720) for relapsing multiple sclerosis. N. Engl. J. Med. 355, 1124–1140 (2006). 16971719 10.1056/NEJMoa052643

[b11] Brinkmann V. . Fingolimod (FTY720): discovery and development of an oral drug to treat multiple sclerosis. Nat. Rev. Drug Discov. 9, 883–897 (2010). 21031003 10.1038/nrd3248

[b12] Labaied M. . Anti-Plasmodium activity of ceramide analogs. Malar. J. 3, 49 (2004). 15588325 10.1186/1475-2875-3-49 PMC539285

[b13] Wang J. R. . Improved sphingolipidomic approach based on ultra-high performance liquid chromatography and multiple mass spectrometries with application to cellular neurotoxicity. Anal. Chem. 86, 5688–5696 (2014). 24844867 10.1021/ac5009964

[b14] Mi J. N., Wang J. R. & Jiang Z. H. Quantitative profiling of sphingolipids in wild Cordyceps and its mycelia by using UHPLC-MS. Sci. Rep. 6, 20870, doi: 10.1038/srep20870 (2016). 26868933 PMC4751452

[b15] Guan X. L. . Biochemical membrane lipidomics during Drosophila development. Dev. Cell. 24, 98–111 (2013). 23260625 10.1016/j.devcel.2012.11.012

[b16] Merrill A. H. Jr. *De novo* sphingolipid biosynthesis: a necessary, but dangerous, pathway. J. Biol. Chem. 277, 25843–25846 (2002). 12011104 10.1074/jbc.R200009200

[b17] Bartke N. & Hannun Y. A. Bioactive sphingolipids: metabolism and function. J. Lipid Res. 50, S91–S96 (2009). 19017611 10.1194/jlr.R800080-JLR200 PMC2674734

[b18] Hla T. & Dannenberg A. Sphingolipid signaling in metabolic disorders. Cell Metabolism 16, 420–434 (2012). 22982021 10.1016/j.cmet.2012.06.017 PMC3466368

[b19] Obeid L. M., Okamoto Y. & Mao C. Yeast sphingolipids: metabolism and biology. Biochim. Biophys. Acta. 1585, 163–171 (2002). 12531550 10.1016/s1388-1981(02)00337-2

[b20] Ternes P. . Two pathways of sphingolipid biosynthesis are separated in the yeast Pichia pastoris. J. Biol. Chem. 286, 11401–11414 (2011). 21303904 10.1074/jbc.M110.193094 PMC3064196

[b21] Merrill A. H. Jr. Sphingolipid and glycosphingolipid metabolic pathways in the era of sphingolipidomics. Chem. Rev. 111, 6387–6422 (2011). 21942574 10.1021/cr2002917 PMC3191729

[b22] Duan J. & Merrill A. H. Jr. 1-Deoxysphingolipids encountered exogenously and made de Novo: Dangerous mysteries inside an Enigma. J. Biol. Chem. 290, 15380–15389 (2015). 25947379 10.1074/jbc.R115.658823 PMC4505451

[b23] Jungalwala F. B., Evans J. E., Kadowaki H. & McCluer R. H. High performance liquid chromatography- chemical ionization mass spectrometry of sphingoid bases usingmoving-belt interface. J. Lipid Res. 25, 209–216 (1984). 6481246

[b24] Penno A. . Hereditary sensory neuropathy type 1 is caused by the accumulation of two neurotoxic sphingolipids. J. Biol. Chem. 285, 11178–11187 (2010). 20097765 10.1074/jbc.M109.092973 PMC2856995

[b25] t’Kindt R. . Profiling and characterizing skin ceramides using reversed-phase liquid chromatography-quadrupole time-of-flight mass spectrometry. Anal. Chem. 84, 403–411 (2012). 22111752 10.1021/ac202646v

[b26] Valsecchi M. . Ceramides as possible nutraceutical compounds: characterization of the ceramides of the Moro blood orange (Citrus sinensis). J. Agric. Food. Chem. 60, 10103–10110 (2012). 22985176 10.1021/jf3027414

[b27] Peñalva D. A. . Atypical surface behavior of ceramides with nonhydroxy and 2-hydroxy very long-chain (C28–C32) PUFAs. Biochim. Biophys. Acta. 1838, 731–738 (2014). 24315999 10.1016/j.bbamem.2013.11.018

[b28] Yang G., Kyoung Seo E., Lee J. H. & Young Lee. Suppression of splenic lymphocyte proliferation by Eucommia ulmoides and genipin. J. Chem. Biodivers. 12, 538–546 (2015). 10.1002/cbdv.201400376 25879499

[b29] Fujita T. . 2-Substituted 2-aminoethanol: Minimum essential structure for immunosuppressive activity of ISP-I (Myriocin). Bioorg. Med. Chem. Lett. 5, 1857–1860 (1995).

[b30] Fujita T. . Fungal metabolites. Part 12. Potent immunosuppressant, 14-deoxomyriocin, (2S,3R,4R)-(E)-2-amino-3,4-dihydroxy-2-hydroxymethyleicos-6-enoic acid and structure-activity relationships of myriocin derivatives. J. Antibiot. 47, 216–224 (1994). 8150718 10.7164/antibiotics.47.216

[b31] Chen J. K., Lane W. S. & Schreiber S. L. The identification of myriocin-binding proteins. Cell Chem. Biol. 6, 221–235 (1999). 10.1016/S1074-5521(99)80038-6 10099133

[b32] Brinkmann V. . The immune modulator FTY720 targets sphingosine 1-phosphate receptors. J. Biol. Chem. 277, 21453–21457 (2002). 11967257 10.1074/jbc.C200176200

[b33] Mandala S. . Alteration of lymphocyte trafficking by sphingosine-1-phosphate receptor agonists. Science. 296, 346–349 (2002). 11923495 10.1126/science.1070238

[b34] Brinkmann V. . Fingolimod (FTY720): discovery and development of an oral drug to treat multiple sclerosis. Nat. Rev. Drug Discov. 9, 883–897 (2010). 21031003 10.1038/nrd3248

