# Supplementary material for: New Immunosuppressive Sphingoid Base and Ceramide Analogues in Wild Cordyceps
Source: Sci Rep. 2016 Dec 14;6:38641. doi: 10.1038/srep38641 (PMC5155214; doi:10.1038/srep38641)

Supplementary Information

New Immunosuppressive Sphingoid Base and Ceramide Analogues in Wild Cordyceps

Jia-Ning Mi, Yuwei Han, Yingqiong Xu, Junping Kou, Jing-Rong Wang and Zhi-Hong Jiang

Table S1. 346 SPLs identified including 171 new SPLs (bolded) from wild Cordyceps by using UHPLC-UHD-Q-TOF-MS/MS.

No.	Name	Formula	RT	Score	Diff (ppm)	Theoretical $[M+H]^+$ (*m/z*)	$[M+H]^+$ (*m/z*)	MS/MS fragment (*m/z*)
1	Sa (d14:0)	C14 H31 N O2	4.99	82.86	0.02	246.2428	246.2430	228.2321, 60.0443
2	So (d14:1)	C14 H29 N O2	4.40	80.77	-2.03	244.2271	244.2275	226.2196, 60.0436
3	So (d14:2)	C14 H27 N O2	3.25	85.66	-1.93	242.2115	242.2115	224.2015, 206.1881, 60.0440
4	So (d14:3)	C14 H25 N O2	2.10	65.07	-0.18	240.1958	240.1958	222.1917, 60.0445
5	So (d15:1)	C15 H31 N O2	6.70	68.91	-1.77	258.2428	258.2429	240.2321, 222.1492
6	So (d16:1)	C16 H33 N O2	7.05	64.91	-2.12	272.2584	272.2585	254.2504, 60.0420
7	Sa (d17:0)	C17 H37 N O2	5.15	99.28	0.80	288.2897	288.2900	270.2799
8	Sa (d18:0)	C18 H39 N O2	6.97	79.10	-2.56	302.3054	302.3052	284.2957, 266.2838, 254.278, 60.0445
9	So (d18:1)	C18 H37 N O2	6.60	90.13	-3.23	300.2897	300.2890	282.2510, 264.2360, 60.0441
**10**	**So (d18:5) ***	**C18 H29 N O2**	**2.67**	**97.56**	**-0.36**	**292.2271**	**292.2272**	**274.2159, 256.2152**
11	So (d19:1)	C19 H39 N O2	8.25	74.08	-1.60	314.3054	314.3049	296.2948, 60.0438
12	So (d19:2)	C19 H37 N O2	6.64	97.44	0.76	312.2897	312.2899	294.2788, 276.2700, 264.2691, 60.0445
13	So (d20:2)	C20 H39 N O2	7.52	76.00	-1.98	326.3054	326.3052	308.2914, 290.2954, 60.0443
**14**	**So (d20:3)**	**C20 H37 N O2**	**7.79**	**97.21**	**0.44**	**324.2897**	**324.2899**	**306.2820, 60.0418**
15	So (d22:1)	C22 H45 N O2	10.64	86.20	-3.88	356.3523	356.3515	338.3456
16	So (d22:1) isomer	C22 H45 N O2	10.17	82.52	-2.07	356.3523	356.3517	338.3203
17	So (d22:2)	C22 H43 N O2	8.60	96.71	0.03	354.3367	354.3366	336.3258
18	So (d22:3)	C22 H41 N O2	7.67	91.82	0.24	352.3210	352.3209	334.3107, 316.3009, 60.0445
**19**	**So (d22:5)**	**C22 H37 N O2**	**6.92**	**63.69**	**-4.81**	**348.2897**	**348.2878**	**330.2419, 60.0445**

**20**	**So (t15:2)**	**C15 H29 N O3**	**3.59**	**98.64**	**-0.37**	**272.2220**	**272.2220**	**254.1970, 60.0445**
**21**	**So (t15:3)**	**C15 H27 N O3**	**2.97**	**72.86**	**-0.61**	**270.2064**	**270.2065**	**252.1951**
22	Sa (t16:0)	C16 H35 N O3	5.12	85.55	-0.02	290.2691	290.2689	272.2527, 254.2401, 236.1139, 242.2471, 60.0438
23	Sa (t18:0)	C18 H39 N O3	6.67	97.40	0.92	318.3003	318.3006	300.2891, 282.2786, 264.2682, 60.0444
24	So (t18:1)	C18 H37 N O3	6.40	97.11	0.52	316.2846	316.2848	298.2741, 280.2635, 262.2529, 250.2535, 60.0445
25	So (t18:2)	C18 H35 N O3	6.87	84.21	1.80	314.2690	314.2697	296.2599, 278.2532, 60.0443
26	So (t19:1)	C19 H39 N O3	7.32	98.97	0.06	330.3003	330.3004	312.2897, 294.2783, 60.0446
**27**	**So (t19:2)**	**C19 H37 N O3**	**6.90**	**98.38**	**-0.29**	**328.2846**	**328.2845**	**310.2738, 298.2743, 292.2637, 280.2638, 274.2572, 262.2534, 60.0444**
28	So (t20:1)	C20 H41 N O3	7.29	69.92	-2.16	344.3159	344.3154	326.2834, 308.2549, 60.0445
**29**	**So (t21:3)**	**C21 H39 N O3**	**9.25**	**88.05**	**-2.07**	**354.3003**	**354.3002**	**336.2901**
**30**	**So (t21:4)**	**C21 H37 N O3**	**7.94**	**72.60**	**1.27**	**352.2846**	**352.2853**	**334.2744, 316.2596**
31	Sa (t22:0)	C22 H47 N O3	8.02	98.41	-1.43	374.3629	374.3624	356.3153, 338.3074, 320.2462
32	So (t22:1)	C22 H45 N O3	8.10	75.99	-2.15	372.3472	372.3466	354.3018, 336.3284
33	So (t22:2)	C22 H43 N O3	7.59	63.48	-2.94	370.3316	370.3312	352.2858, 334.2853, 316.2329, 60.0444
34	So (t23:4)	C23 H41 N O3	9.50	74.92	1.12	380.3159	380.3161	362.3060
35	So (m14:3)	C14 H25 N O	3.72	84.63	-0.95	224.2009	224.2014	206.1902
36	Sa (m17:0)	C17 H37 N O	6.74	86.06	1.37	272.2948	272.2952	254.2502
37	Sa (m18:0)	C18 H39 N O	7.20	98.31	-1.73	286.3104	286.3110	268.2989
38	So (m18:1)	C18 H37 N O	6.95	83.22	0.68	284.2948	284.2948	266.2868
**39**	**So (m22:1)**	**C22 H45 N O**	**11.12**	**96.58**	**0.53**	**340.3574**	**340.3577**	**322.3447**
**40**	**So (m22:2)**	**C22 H43 N O**	**9.47**	**99.21**	**-0.76**	**338.3417**	**338.3416**	**320.3321**
**41**	**So (m22:3)**	**C22 H41 N O**	**7.60**	**98.61**	**0.64**	**336.3261**	**336.3263**	**318.3153**
**42**	**So (m22:3) isomer**	**C22 H41 N O**	**9.92**	**84.94**	**0.03**	**336.3261**	**336.3261**	**318.3134**

43	Sphingofungin A	C21 H41 N3 O6	9.34	89.37	-1.50	432.3068	432.3064	414.3447, 396.3327, 378.3281
**44**	**Cer (m14:2/18:2)**	**C32 H57 N O2**	**10.78**	**88.80**	**-3.12**	**488.4462**	**488.4452**	**470.3669, 226.2164, 208.2063, 196.2068, 44.0482**
**45**	**Cer (m14:3/18:2)**	**C32 H55 N O2**	**10.52**	**95.97**	**-0.47**	**486.4306**	**486.4305**	**468.4108, 224.2015, 206.1907, 194.1904, 44.0483**
**46**	**Cer (m14:3/18:2) isomer**	**C32 H55 N O2**	**9.27**	**79.44**	**-5.25**	**486.4306**	**486.4282**	**468.3868, 206.1906**
**47**	**Cer (m14:3/24:1)**	**C38 H69 N O2**	**14.27**	**96.33**	**-1.41**	**572.5401**	**572.5395**	**554.5282, 224.2013, 206.1909, 194.1909, 44.0484**
**48**	**Cer (m15:1/5:0)**	**C20 H39 N O2**	**8.55**	**99.05**	**1.22**	**326.3054**	**326.3058**	**308.2948, 224.2011**
**49**	**Cer (m15:2/18:2)**	**C33 H59 N O2**	**11.68**	**98.70**	**1.10**	**502.4619**	**502.4613**	**484.4546, 240.2323, 222.2219, 210.2215, 44.0483**
**50**	**Cer (m15:3/18:1)**	**C33 H59 N O2**	**11.40**	**90.67**	**-1.07**	**502.4619**	**502.4617**	**484.4170, 238.2164, 220.2065, 208.2060, 44.0482**
**51**	**Cer (m16:3/18:1)**	**C34 H61 N O2**	**11.88**	**86.43**	**-3.35**	**516.4775**	**516.4757**	**498.4709, 252.2318, 234.2216, 222.2223, 44.0483**
**52**	**Cer (m16:3/22:1)**	**C38 H69 N O2**	**14.68**	**76.67**	**1.40**	**572.5401**	**572.5403**	**554.5358, 252.2135, 234.2220, 222.2222, 44.0483**
**53**	**Cer (m18:2/3:0)**	**C21 H39 N O2**	**7.82**	**99.01**	**-0.02**	**338.3054**	**338.3050**	**264.2679**
**54**	**Cer (m18:2/19:0)**	**C37 H71 N O2**	**15.28**	**95.01**	**-0.75**	**562.5558**	**562.5555**	**544.5445, 282.2789, 264.2681, 252.2704, 44.0484**
**55**	**Cer (m18:3/18:1)**	**C36 H65 N O2**	**12.98**	**97.15**	**-0.21**	**544.5088**	**544.5088**	**526.4965, 280.2627, 262.2525, 250.2529, 44.0483**
**56**	**Cer (m18:3/18:2)**	**C36 H63 N O2**	**12.22**	**94.15**	**-0.39**	**542.4932**	**542.4931**	**524.4831, 280.2628, 262.2521, 250.2531, 44.0483**
**57**	**Cer (m18:5/5:0)**	**C23 H37 N O2**	**7.53**	**99.45**	**0.87**	**360.2897**	**360.2900**	**342.2793, 258.2229**
**58**	**Cer (m19:4/16:0)**	**C35 H63 N O2**	**10.78**	**97.11**	**-1.79**	**530.4932**	**530.4923**	**512.4818, 292.2627, 274.2528, 262.2527, 44.0482**
**59**	**Cer (m19:3/17:0)**	**C35 H65 N O2**	**13.43**	**95.84**	**-0.78**	**532.5088**	**532.5093**	**514.4972, 294.2784, 276.2681, 264.2681, 44.0483**
60	Cer (d14:0/16:0)	C30 H61 N O3	11.25	93.31	1.77	484.4724	484.4710	466.4612, 448.4499, 246.2411, 228.2317, 210.2203, 198.2203
61	Cer (d14:0/18:0)	C32 H65 N O3	12.47	96.42	-1.61	512.5037	512.5031	494.4912, 476.4834, 246.2453, 228.2326, 210.2220, 198.2216, 60.0445
**62**	**Cer (d14:0/18:1)**	**C32 H63 N O3**	**11.67**	**84.13**	**-3.03**	**510.4881**	**510.4865**	**492.4745, 474.4684, 462.3501, 246.2431, 228.2322, 210.2218, 198.2218, 60.0444**
63	Cer (d14:0/20:0)	C34 H69 N O3	13.68	98.35	-0.79	540.5350	540.5453	522.5236, 504.5230, 246.2427, 228.2325, 210.2221, 198.2220, 60.0445
**64**	**Cer (d14:0/22:0)**	**C36 H73 N O3**	**15.08**	**99.04**	**-0.34**	**568.5663**	**568.5661**	**550.5549, 532.5438, 246.2438, 228.2325, 210.2220, 198.2221, 60.0444**

**65**	**Cer (d14:0/23:0)**	**C37 H75 N O3**	**15.77**	**95.74**	**-1.36**	**582.5820**	**582.5813**	**564.5697, 546.5581, 246.2430, 228.2323, 210.2217, 198.2223, 60.0444**
66	Cer (d14:0/24:0)	C38 H77 N O3	16.38	96.05	-0.79	596.5976	596.5982	578.5862, 560.5761, 246.2435, 228.2324, 210.2222, 198.2221, 60.0444
67	Cer (d16:0/20:0)	C36 H73 N O3	15.06	98.41	-1.11	568.5663	568.5669	550.5550, 532.5435, 520.5305, 274.2729, 256.2626, 238.2517, 226.2522, 60.0439
**68**	**Cer (d16:0/22:0)**	**C38 H77 N O3**	**16.40**	**97.75**	**-0.80**	**596.5976**	**596.5973**	**578.5862, 560.5761, 274.2731, 256.2637, 238.2533, 226.253, 60.0444**
**69**	**Cer (d16:0/23:0)**	**C39 H79 N O3**	**17.02**	**89.50**	**-2.81**	**610.6133**	**610.6120**	**592.6018, 574.5929, 274.2718, 256.2638, 238.2528, 226.2522, 60.0444**
70	Cer (d18:0/16:0)	C34 H69 N O3	13.50	98.72	-0.27	540.5350	540.5349	522.5236, 504.5130, 302.3046, 284.2944, 266.2841, 254.2839, 60.0445
71	Cer (d18:0/17:0)	C35 H71 N O3	14.30	63.16	7.99	554.5507	554.5472	536.5386, 518.5276, 302.3016, 284.2929, 266.2833, 60.0438
72	Cer (d18:0/18:0)	C36 H73 N O3	15.00	98.27	-1.19	568.5663	568.5669	550.5544, 532.5438, 302.3084, 284.2946, 266.2841, 254.2842, 60.0444
73	Cer (d18:0/18:1)	C36 H71 N O3	14.85	95.87	-0.83	566.5507	566.5511	302.3084, 284.2948, 266.2843, 254.2842, 60.0445
74	Cer (d18:0/18:2)	C36 H69 N O3	13.98	65.50	-2.34	564.5350	564.5348	546.5240, 528.5161, 516.5123, 284.2947, 266.2841, 254.2837, 60.0444
75	Cer (d18:0/20:0)	C38 H77 N O3	16.60	96.69	-0.81	596.5976	596.5982	578.5863, 560.5765, 302.3031, 284.2945, 266.2836, 60.0437
76	Cer (d18:0/22:0) isomer	C40 H81 N O3	17.72	95.99	-2.10	624.6289	624.6277	606.6163, 588.6055, 302.3052, 284.2949, 266.2838, 254.2844, 60.0444
77	Cer (d18:0/22:0)	C40 H81 N O3	17.97	89.99	-3.09	624.6289	624.6275	606.6174, 588.6058, 302.3078, 284.2948, 266.2840, 254.2833, 60.0444
78	Cer (d18:0/23:0)	C41 H83 N O3	18.55	85.94	-3.16	638.6446	638.6433	620.6329, 602.6228, 302.3032, 284.2955, 266.2831, 254.2871, 60.0444
79	Cer (d18:0/24:0)	C42 H85 N O3	19.40	92.59	-2.90	652.6602	652.6588	634.6487, 616.6352, 302.3084, 284.2948, 266.2841, 254.2843, 60.0445
80	Cer (d18:0/24:1)	C42 H83 N O3	17.96	89.09	-0.90	650.6446	650.6431	632.6302, 614.6142, 302.3016, 284.2921, 266.2827, 254.2727, 60.0439
81	Cer (d18:0/25:0)	C43 H87 N O3	20.20	88.62	-3.50	666.6759	666.6737	648.6634, 630.6514, 302.3073, 284.2943, 266.2824, 254.2831, 60.0444
82	Cer (d18:0/26:0)	C44 H89 N O3	21.00	90.71	-2.91	680.6915	680.6901	662.6796, 644.6697, 302.3083, 284.2947, 266.2844, 254.2847, 60.0444
**83**	**Cer (d20:0/25:0)**	**C45 H91 N O3**	**21.84**	**82.72**	**-3.64**	**694.7072**	**694.7056**	**676.6960, 658.6845, 330.3371, 312.3255, 294.3154, 282.3139, 60.0445**
84	Cer (d20:0/26:0)	C46 H93 N O3	22.40	91.72	-2.57	708.7228	708.7216	690.7108, 672.6992, 660.6947, 330.3376, 312.3259, 294.3168, 282.3158, 60.0444
**85**	**Cer (d22:0/25:0)**	**C47 H95 N O3**	**22.90**	**87.17**	**-3.46**	**722.7385**	**722.7365**	**704.7256, 686.7147, 358.3704, 340.3566, 322.3473, 310.3485, 60.0445**

**86**	**Cer (d22:0/26:0)**	**C48 H97 N O3**	**23.29**	**94.06**	**-2.63**	**736.7541**	**736.7526**	**718.7423, 700.7305, 358.3706, 340.3573, 322.3466, 60.0444**
**87**	**Cer (d24:0/26:0)**	**C50 H101 N O3**	**24.22**	**83.57**	**-1.74**	**764.7854**	**764.7829**	**746.7729, 728.7612, 396.4187, 386.4028, 368.3890, 350.3746, 338.3770, 60.0444**
**88**	**Cer (d25:0/24:0)**	**C49 H99 N O3**	**23.75**	**88.87**	**-3.37**	**750.7698**	**750.7671**	**732.7573, 714.7453, 382.4034, 364.3927, 352.3752, 60.0442**
89	Cer (d18:0/16:0(OH))	C34 H69 N O4	13.17	70.07	-2.68	556.5299	556.5290	538.5192, 520.5085, 502.4947, 490.4969, 302.3046, 284.2952, 266.2845, 254.2844, 60.0444
90	Cer (d18:0/20:0(OH))	C38 H77 N O4	15.75	64.18	-4.15	612.5925	612.5923	594.5814, 576.5713, 558.5542, 302.3026, 284.2956, 266.2837, 254.2809, 60.0444
91	Cer (d18:0/24:0(OH))	C42 H85 N O4	18.57	93.69	-1.60	668.6551	668.6543	650.6424, 632.6333, 614.6246, 602.6232, 302.3037, 284.2948, 266.2842, 254.284, 60.0444
92	Cer (d20:0/26:0(OH))	C46 H93 N O4	21.87	86.66	-2.04	724.7177	724.7167	706.7041, 688.6884, 312.3260, 294.3125, 60.0446
**93**	**Cer (d22:0/25:0(OH))**	**C47 H95 N O4**	**22.40**	**81.20**	**-3.67**	**738.7334**	**738.7309**	**720.7174, 702.7103, 684.5929, 340.3558, 322.3416, 310.3420, 60.044**
**94**	**Cer (t14:0/14:0)**	**C28 H57 N O4**	**9.32**	**97.85**	**-1.34**	**472.4360**	**472.4356**	**454.3674, 436.3568, 208.2068, 60.0443**
95	Cer (t18:0/16:0)	C34 H69 N O4	12.48	96.83	2.11	556.5299	556.5288	538.5185, 520.5069, 318.2989, 300.2874, 282.2780, 264.2680, 252.2713, 60.0438
96	Cer (t18:0/16:1)	C34 H67 N O4	12.66	74.58	-1.17	554.5143	554.5137	536.5047, 518.4890, 318.3003, 300.2896, 282.2795, 264.2687, 252.2690, 60.0445
97	Cer (t18:0/17:0)	C35 H71 N O4	13.11	73.70	1.17	570.5456	570.5451	552.5263, 534.5221, 318.3001, 300.2903, 282.2794, 264.2681, 252.2698, 60.0445
**98**	**Cer (t18:0/17:1)**	**C35 H69 N O4**	**13.20**	**72.43**	**1.82**	**568.5299**	**568.5300**	**550.5195, 532.5077, 514.4987, 502.4990, 318.2974, 300.2925, 282.2794, 264.2682, 252.2678, 60.0444**
99	Cer (t18:0/18:0)	C36 H73 N O4	13.75	98.04	-0.38	584.5612	584.5611	566.5493, 548.5379, 530.5316, 518.5301, 318.3004, 300.2895, 282.2794, 264.2686, 252.2691, 60.0445

100	Cer (t18:0/18:1)	C36 H71 N O4	13.94	80.22	0.11	582.5456	582.5456	564.5340, 546.5215, 528.5087, 318.3002, 300.2893, 282.2791, 264.2685, 252.2687, 60.0444
101	Cer (t18:0/18:2)	C36 H69 N O4	12.15	96.38	2.16	580.5299	580.5287	562.5163, 544.5061, 526.4970, 318.2994, 300.2886, 282.2788, 264.2679, 252.2683, 60.0439
**102**	**Cer (t18:0/19:1)**	**C37 H73 N O4**	**14.46**	**72.71**	**-1.24**	**596.5612**	**596.5607**	**578.5343, 560.5363, 542.4854, 318.3007, 300.2921, 282.2795, 264.2686, 252.2690, 60.0446**
103	Cer (t18:0/20:0)	C38 H77 N O4	15.10	95.07	-0.50	612.5925	612.5923	594.5811, 576.5722, 558.5618, 318.3011, 300.2898, 282.2791, 264.2686, 252.2689, 60.0445
104	Cer (t18:0/20:1)	C38 H75 N O4	15.10	80.53	-3.03	610.5769	610.5751	592.5672, 574.5573, 318.2991, 300.2888. 282.2785, 264.2679, 252.2692, 60.0438
105	Cer (t18:0/21:0)	C39 H79 N O4	15.77	95.31	-1.10	626.6082	626.6077	608.5954, 590.5868, 572.5736, 560.5743, 318.3002, 300.2892, 282.2791, 264.2684, 252.2689, 60.0444
**106**	**Cer (t18:0/21:1)**	**C39 H77 N O4**	**15.70**	**75.08**	**-1.99**	**624.5925**	**624.5938**	**606.5831, 588.5529, 570.4213, 318.3005, 300.2898, 282.2793, 264.2687, 252.2690, 60.0445**
107	Cer (t18:0/22:0)	C40 H81 N O4	16.43	99.02	0.03	640.6238	640.6238	622.6119, 604.6010, 586.5907, 574.5893, 318.3004, 300.2897, 282.2795, 264.2687, 252.2691, 60.0445
108	Cer (t18:0/22:1)	C40 H79 N O4	16.30	87.99	0.88	638.6082	638.6060	620.5993, 602.5904, 318.3001, 300.2894, 282.2794, 264.2686, 252.2690, 60.0445
109	Cer (t18:0/23:0)	C41 H83 N O4	17.25	98.64	-0.15	654.6395	654.6395	636.6284, 618.6171, 600.6059, 318.3009, 300.2895, 282.2797, 264.2688, 252.2691, 60.0445
110	Cer (t18:0/23:1)	C41 H81 N O4	17.00	80.56	1.78	652.6238	652.6233	634.6136, 616.5993, 598.5534, 318.2992, 300.2883, 282.2796, 264.2685, 252.2680, 60.0439
111	Cer (t18:0/24:0)	C42 H85 N O4	17.85	98.59	-0.31	668.6551	668.6549	650.6435, 632.6327, 614.6206, 602.6235, 318.3003, 300.2897,

								282.2796, 264.2688, 252.2688, 60.0445
112	Cer (t18:0/24:1)	C42 H83 N O4	17.40	70.87	1.23	666.6395	666.6383	648.6237, 630.6147, 318.2999, 300.2893, 282.2791, 264.2683, 252.2688, 60.0444
**113**	**Cer (t18:0/24:2)**	**C42 H81 N O4**	**15.68**	**90.97**	**2.65**	**664.6238**	**664.6221**	**646.5980, 628.5949, 610.5137, 406.3669, 364.3572, 318.2947, 300.2905, 282.2787, 264.2682, 252.2680, 60.0438**
**114**	**Cer (t18:0/24:5)**	**C42 H75 N O4**	**16.05**	**80.65**	**-1.08**	**658.5769**	**658.5750**	**640.5572, 622.4898, 604.5243, 400.3674, 358.3593, 318.3116, 300.2900, 282.2790, 264.2683, 252.2692, 60.0438**
115	Cer (t18:0/25:0)	C43 H87 N O4	18.65	93.89	-1.80	682.6708	682.6698	664.6592, 646.6489, 628.6402, 318.3007, 300.2895, 282.2792, 264.2688, 252.2687, 60.0445
116	Cer (t18:0/26:0) isomer	C44 H89 N O4	18.77	96.22	-2.20	696.6864	696.6850	678.6762, 660.6663, 642.6543, 630.6419, 318.2998, 300.2892, 282.2793, 264.2686, 252.2674, 60.0444
117	Cer (t18:0/27:0) isomer	C45 H91 N O4	19.33	91.39	1.28	710.7021	710.7045	692.6832, 674.6883, 300.2912, 282.2786, 264.2683, 60.0444
118	Cer (t18:0/26:0)	C44 H89 N O4	19.48	96.19	-2.22	696.6864	696.6850	678.6746, 660.6626, 318.3009, 300.2890, 282.2792, 264.2694, 252.2681, 60.0444
119	Cer (t18:0/27:0)	C45 H91 N O4	20.35	86.91	-3.12	710.7021	710.7004	692.6925, 674.6793, 656.6145, 318.3012, 300.2883, 282.2791, 264.2674, 60.0446
120	Cer (t18:0/25:1)	C43 H85 N O4	18.28	80.66	1.88	680.6551	680.6541	662.6531, 644.6406, 318.2999, 300.2890, 282.2793, 264.2682, 252.2694, 60.0444
121	Cer (t18:0/26:1)	C44 H87 N O4	18.58	76.77	-4.31	694.6708	694.6677	676.6412, 658.6339, 640.6337, 318.2991, 300.2894, 282.2789, 264.2680, 252.2414, 60.0439
**122**	**Cer (t18:0/26:5)**	**C44 H79 N O4**	**16.05**	**61.51**	**7.15**	**686.6082**	**686.6039**	**668.6074, 650.6051, 632.5979, 318.2995, 300.2918, 282.2780, 264.2683, 60.0438**
123	Cer (t19:0/18:1)	C37 H73 N O4	14.50	70.45	3.42	596.5612	596.5602	578.5495, 560.5412, 548.5348, 332.3153, 314.3049, 296.2946,

								278.2842, 266.2574, 60.0438
124	Cer (t19:0/18:2)	C37 H71 N O4	13.60	72.83	1.21	594.5456	594.5451	576.5337, 558.5162, 332.3162, 314.3056, 296.2948, 278.2834, 266.2282, 60.0440
125	Cer (t20:0/26:0)	C46 H93 N O4	21.24	96.16	-2.32	724.7177	724.7161	706.7055, 688.6957, 346.3329, 328.3209, 310.3114, 292.2996, 280.2978, 60.0444
**126**	**Cer (t20:0/26:1)**	**C46 H91 N O4**	**20.72**	**63.46**	**-1.85**	**722.7021**	**722.7009**	**704.7011, 686.6849, 346.3299, 328.3222, 310.3095, 292.2997, 280.3009, 60.0445**
**127**	**Cer (t20:0/30:1)**	**C50 H99 N O4**	**22.40**	**83.42**	**-3.29**	**778.7647**	**778.7627**	**760.7500, 742.7370, 310.3111, 292.2998, 280.2998, 60.0445**
**128**	**Cer (t20:0/31:1)**	**C51 H101 N O4**	**23.27**	**75.18**	**-4.77**	**792.7803**	**792.7782**	**774.7702, 756.7553, 738.7353, 310.3082, 292.2988, 280.3001, 60.0444**
129	Cer (t22:0/25:0)	C47 H95 N O4	21.90	94.15	-2.71	738.7334	738.7316	720.7216, 702.7125, 374.3682, 356.3524, 338.3409, 320.3314, 308.3336, 60.0444
130	Cer (t22:0/26:0)	C48 H97 N O4	22.44	95.47	-2.37	752.749	752.7474	734.7371, 716.7219, 698.7067, 374.3607, 356.3535, 338.3418, 320.3323, 308.3328, 60.0445
**131**	**Cer (t22:0/26:1)**	**C48 H95 N O4**	**22.47**	**61.42**	**0.05**	**750.7334**	**750.7345**	**732.7208, 714.7138, 374.3637, 356.3513, 338.3410, 320.3314, 308.3287, 60.0444**
132	Cer (t22:0/27:0)	C49 H99 N O4	22.92	93.63	-2.63	766.7647	766.7630	748.7530, 730.7428, 374.3635, 356.3529, 338.3420, 320.3311, 308.3214, 60.0443
133	Cer (t22:0/28:0)	C50 H101 N O4	23.50	95.82	-1.75	780.7803	780.7786	762.7694, 744.7563, 726.7574, 374.3592, 356.3517, 338.3406, 320.3308, 308.3273, 60.0445
**134**	**Cer (t16:0/12:0(OH))**	**C28 H57 N O5**	**7.67**	**80.92**	**-5.28**	**488.4310**	**488.4285**	**470.3743, 236.2407, 60.0443**
135	Cer (t18:0/16:0(OH))	C34 H69 N O5	12.05	91.03	-2.65	572.5249	572.5237	554.4728, 542.4728, 536.5024, 518.4919, 500.4885, 318.2993, 300.2893, 282.2792, 264.2683, 252.2691, 60.0440
**136**	**Cer (t18:0/17:0(OH))**	**C35 H71 N O5**	**12.61**	**65.94**	**3.03**	**586.5405**	**586.5392**	**568.5296, 550.5165, 532.5129, 318.2998, 300.2871, 282.2783,**

								**264.2678, 60.0438**
**137**	**Cer (t18:0/17:4(OH))**	**C35 H63 N O5**	**10.20**	**85.77**	**-0.49**	**578.4779**	**578.4774**	**560.4748, 300.2878, 282.2789, 264.2681, 252.2725, 60.0444**
138	Cer (t18:0/18:0(OH))	C36 H63 N O5	13.20	96.03	-0.08	600.5562	600.5565	582.5444, 564.5341, 546.5231, 528.5074, 318.2988, 300.2889, 282.2786, 264.2679, 252.2680, 60.0438
139	Cer (t18:0/20:0(OH))	C38 H77 N O5	14.50	82.50	-3.27	628.5875	628.5857	610.5772, 592.5657, 574.5547, 318.2995, 300.2892, 282.2792, 264.2683, 252.2689, 60.0444
140	Cer (t18:0/21:0(OH))	C39 H79 N O5	15.15	93.93	0.48	642.6031	642.6031	624.5913, 606.5805, 588.5660, 570.4251, 318.3165, 300.2898, 282.2783, 264.2678, 252.2670, 60.0438
141	Cer (t18:0/22:0(OH))	C40 H81 N O5	15.80	99.55	-0.17	656.6188	656.6186	638.6073, 620.5966, 602.5860, 584.5751, 318.3005, 300.2900, 282.2798, 264.2691, 252.2691, 60.0445
142	Cer (t18:0/23:0(OH))	C41 H83 N O5	16.45	99.00	0.13	670.6344	670.6345	652.6223, 634.6119, 616.6004, 604.5986, 598.5930, 318.3001, 300.2897, 282.2796, 264.2688, 252.2690, 60.0445
143	Cer (t18:0/23:1(OH))	C41 H81 N O5	15.32	94.48	-0.67	668.6188	668.6185	650.6076, 632.5961, 614.5852, 596.5374, 318.3008, 300.2895, 282.2794, 264.2685, 252.2694, 60.0444
144	Cer (t18:0/24:0(OH))	C42 H85 N O5	17.13	98.90	-0.24	684.6501	684.6497	666.6391, 648.6288, 630.6174, 612.6071, 318.3006, 300.2902, 282.2798, 264.2692, 252.2693, 60.0445
145	Cer (t18:0/24:1(OH))	C42 H83 N O5	16.02	97.11	-1.56	682.6344	682.6333	664.6214, 646.6117, 628.6013, 610.5933, 318.3001, 300.2894, 282.2796, 264.2688, 252.2686, 60.0445
146	Cer (t18:0/25:0(OH))	C43 H87 N O5	17.83	98.68	-0.38	698.6657	698.6653	680.6533, 662.6426, 644.6315, 626.6226, 614.6232, 318.3002, 300.2894, 282.2795, 264.2686, 252.2687, 60.0444
147	Cer (t18:0/26:0(OH))	C44 H89 N O5	18.58	95.18	-1.79	712.6814	712.6802	694.6694, 676.6593, 658.6466, 640.6314, 318.3008, 300.2892, 282.2794, 264.2685, 252.2697, 60.0444
148	Cer (d14:1/16:0)	C30 H59 N O3	11.00	85.53	-2.51	482.4568	482.4561	226.2164, 208.2064, 196.2068, 60.0444

149	Cer (d14:1/18:0)	C32 H63 N O3	12.10	70.12	3.25	510.4881	510.4866	244.1701, 226.2159, 208.2064, 196.2065, 60.0443
150	Cer (d14:1/18:1)	C32 H61 N O3	11.32	75.17	-1.78	508.4724	508.4715	490.4637, 244.1489, 226.2164, 208.2065, 196.2068, 60.0443
**151**	**Cer (d14:1/18:2)**	**C32 H59 N O3**	**11.16**	**91.52**	**-2.46**	**506.4568**	**506.4553**	**488.4472, 470.6828, 226.2163, 208.2048, 196.2048, 60.0438**
152	Cer (d14:1/20:0)	C34 H67 N O3	13.30	60.57	2.67	538.5194	538.5200	520.5080, 502.4976, 490.4968, 226.2163, 208.2055, 196.2052, 60.0445
**153**	**Cer (d14:1/20:4)**	**C34 H59 N O3**	**11.68**	**79.05**	**2.68**	**530.4568**	**530.4548**	**512.4422, 226.2152, 208.2064, 196.2070, 60.0442**
154	Cer (d14:1/22:0)	C36 H71 N O3	14.63	98.78	0.32	566.5507	566.5507	548.5370, 530.5258, 226.2165, 208.2067, 196.2065, 60.0445
155	Cer (d14:1/23:0)	C37 H73 N O3	15.30	97.85	-0.99	580.5663	580.5664	562.5615, 544.5448, 226.2158, 208.2063, 196.2063, 60.0444
156	Cer (d14:1/24:0)	C38 H75 N O3	16.03	85.79	0.26	594.5820	594.5817	576.5717, 558.5551, 546.5523, 226.2164, 208.2067, 196.2065, 60.0445
157	Cer (d14:2/16:0)	C30 H57 N O3	10.78	94.25	2.38	480.4411	480.4400	462.3710, 224.2009, 206.1906, 194.1890, 60.0445
**158**	**Cer (d14:2/18:2)**	**C32 H57 N O3**	**11.06**	**77.82**	**3.59**	**504.4411**	**504.4390**	**242.1975, 224.1959, 206.1905, 194.1914, 60.0443**
**159**	**Cer (d14:2/20:4)**	**C34 H57 N O3**	**10.96**	**85.99**	**3.65**	**528.4411**	**528.4393**	**510.4281, 224.2026, 206.1893, 60.0438**
**160**	**Cer (d14:2/20:5)**	**C34 H55 N O3**	**10.48**	**86.16**	**4.11**	**526.4255**	**526.4235**	**508.4086, 206.1894, 60.0437**
**161**	**Cer (d14:2/20:6)**	**C34 H53 N O3**	**10.13**	**80.80**	**3.88**	**524.4098**	**524.4069**	**506.3812, 224.2058, 206.1891, 60.0437**
**162**	**Cer (d14:2/24:3)**	**C38 H67 N O3**	**14.15**	**88.75**	**-3.97**	**586.5194**	**586.5169**	**568.5049, 538.4920, 224.2005, 206.1909, 194.1884, 60.0444**
**163**	**Cer (d14:2/25:3)**	**C39 H69 N O3**	**14.82**	**80.84**	**-4.03**	**600.5350**	**600.5324**	**582.5183, 552.5139, 224.2065, 206.1907, 194.1917, 60.0444**
**164**	**Cer (d14:2/26:3)**	**C40 H71 N O3**	**15.53**	**88.32**	**-3.66**	**614.5507**	**614.5484**	**596.5388, 566.5217, 224.2010, 206.1905, 194.1142, 60.0444**
165	Cer (d15:1/20:0)	C35 H69 N O3	13.88	73.53	-0.17	552.5350	552.5341	534.5190, 504.5190, 240.2322, 222.2219, 210.2216, 60.0444
166	Cer (d15:1/22:0)	C37 H73 N O3	15.08	87.15	-0.67	580.5663	580.5656	562.5619, 544.5423, 240.2324, 222.2221, 210.2222, 60.0444
**167**	**Cer (d15:1/22:1)**	**C37 H71 N O3**	**14.68**	**75.69**	**-1.61**	**578.5507**	**578.5499**	**560.5363, 542.5317, 530.5252, 240.2324, 222.2220, 210.2218, 60.0444**
**168**	**Cer (d15:2/22:1)**	**C37 H69 N O3**	**14.00**	**69.76**	**-1.63**	**576.5350**	**576.5342**	**558.5212, 238.2157, 220.2064, 208.2057, 60.0444**
169	Cer (d16:1/22:0)	C38 H75 N O3	15.83	93.75	0.01	594.5820	594.5818	576.5789, 558.5592, 546.5678, 272.2603, 254.2479, 236.2373, 224.2377, 60.0444

170	Cer (d16:1/22:1)	C38 H73 N O3	15.21	80.88	2.30	592.5663	592.5669	574.5528, 556.5449, 254.2283, 236.2364, 224.2380, 60.0438
**171**	**Cer (d16:1/22:2)**	**C38 H71 N O3**	**15.07**	**62.36**	**0.33**	**590.5507**	**590.5485**	**572.5410, 272.2396, 254.2482, 236.2374, 224.2377, 60.0444**
172	Cer (d16:1/24:0)	C40 H79 N O3	17.21	93.81	0.54	622.6133	622.6125	604.6023, 586.5933, 574.5919, 254.2468, 236.2370, 224.2236, 60.0439
**173**	**Cer (d16:2/22:2)**	**C38 H69 N O3**	**14.61**	**77.60**	**4.60**	**588.5350**	**588.5335**	**570.5212, 252.2318, 234.2216, 222.2207, 60.0439**
**174**	**Cer (d16:2/23:1)**	**C39 H73 N O3**	**15.53**	**80.53**	**3.43**	**604.5663**	**604.5646**	**586.5535, 568.5508, 252.2267, 234.2215, 222.2207, 60.0438**
**175**	**Cer (d16:2/23:2)**	**C39 H71 N O3**	**15.28**	**86.09**	**3.94**	**602.5507**	**602.5485**	**584.5293, 252.2321, 234.2216, 222.2217, 60.0444**
**176**	**Cer (d16:2/24:2)**	**C40 H73 N O3**	**16.05**	**75.32**	**3.60**	**616.5663**	**616.5644**	**598.5288, 252.2318, 234.2220, 222.2215, 60.0445**
177	Cer (d18:1/14:1)	C32 H61 N O3	11.56	88.80	-3.29	508.4724	508.4722	300.2695, 282.2791, 264.2684, 252.2678, 60.0443
178	Cer (d18:1/16:0)	C34 H67 N O3	13.20	92.16	2.67	538.5194	538.5200	520.5080, 502.4976, 490.4968, 300.2920, 282.2788, 264.2682, 252.2681, 60.0438
179	Cer (d18:1/16:1)	C34 H65 N O3	12.70	94.00	-0.28	536.5037	536.5037	518.4883, 500.4830, 488.4807, 300.2890, 282.2789, 264.2688, 252.2689, 60.0444
180	Cer (d18:1/17:0)	C35 H69 N O3	13.80	80.51	-0.37	552.5350	552.5345	534.5235, 516.5156, 504.5099, 282.2786, 264.2680, 252.2697, 60.0438
181	Cer (d18:1/17:1)	C35 H67 N O3	13.35	94.17	0.45	550.5194	550.5194	532.5075, 520.5060, 514.4981, 300.2866, 282.2784, 264.2683, 60.0439
182	Cer (d18:1/18:0)	C36 H71 N O3	14.46	98.8	-1.01	566.5507	566.5512	548.5392, 530.5392, 300.2905, 282.2787, 264.2683, 252.2691, 60.0437
183	Cer (d18:1/18:1)	C36 H69 N O3	13.95	90.93	-0.81	564.5350	564.5355	546.5240, 528.5261, 300.2706, 282.2789, 264.2685, 252.2689, 60.0444
184	Cer (d18:1/18:2)	C36 H67 N O3	13.50	99.22	-0.60	562.5194	562.5197	544.5092, 526.4973, 282.2799, 264.2687, 252.2134, 60.0445
185	Cer (d18:1/20:0)	C38 H75 N O3	15.84	94.73	-0.43	594.5820	594.5823	576.5726, 558.5505, 300.2866, 282.2785, 264.2683, 252.2677, 60.0445
186	Cer (d18:1/20:1)	C38 H73 N O3	15.23	75.43	-0.45	592.5663	592.5668	574.5589, 556.5482, 300.2693, 282.2785, 264.2677, 252.2622, 60.0438
187	Cer (d18:1/21:0)	C39 H77 N O3	16.55	72.25	1.68	608.5976	608.5974	590.5844, 572.5753, 300.2740, 282.2788, 264.2683, 252.2695, 60.0433
188	Cer (d18:1/22:0)	C40 H79 N O3	17.25	65.73	0.12	622.6133	622.6122	604.5901, 586.5897, 574.5606, 300.2874, 282.2793, 264.2685, 252.2692, 60.0443
189	Cer (d18:1/22:1)	C40 H77 N O3	16.53	75.34	-3.54	620.5976	620.5969	602.5862, 584.5745, 300.2867, 282.2782, 264.2676, 252.2681, 60.0438

190	Cer (d18:1/22:2)	C40 H75 N O3	16.32	61.25	-3.06	618.5820	618.5790	600.5703, 582.5579, 300.2832, 282.2801, 264.2685, 252.2690, 60.0444
191	Cer (d18:1/23:0)	C41 H81 N O3	17.95	68.37	-1.02	636.6289	636.6282	618.6271, 600.6136, 588.6035, 300.2935, 282.2794, 264.2686, 252.2680, 60.0445
192	Cer (d18:1/23:1)	C41 H79 N O3	17.02	66.13	0.08	634.6133	634.6129	616.5984, 598.5948, 300.2891, 282.2786, 264.2688, 252.2680, 60.0444
**193**	**Cer (d18:1/23:5)**	**C41 H71 N O3**	**13.00**	**75.79**	**-3.03**	**626.5507**	**626.5537**	**608.5416, 590.5322, 300.2876, 282.2781, 264.2680, 252.2646, 60.0438**
194	Cer (d18:1/24:0)	C42 H83 N O3	18.68	70.23	-0.73	650.6446	650.6431	632.6388, 614.6308, 602.6239, 300.2876, 282.2794, 264.2686, 252.2689, 60.0444
195	Cer (d18:1/24:1)	C42 H81 N O3	17.81	64.02	1.52	648.6289	648.6287	630.6203, 612.6077, 600.6053, 300.2895, 282.2793, 264.2688, 252.2689, 60.0444
196	Cer (d18:1/24:2)	C42 H79 N O3	17.59	70.45	-2.54	646.6133	646.6106	628.6036, 610.5821, 282.2786, 264.2679, 252.2686, 60.0439
197	Cer (d18:1/24:5)	C42 H73 N O3	13.60	83.53	-2.66	640.5663	640.5683	622.5577, 604.5419, 300.2869, 282.2816, 264.2679, 252.2646, 60.0438
198	Cer (d18:1/25:0)	C43 H85 N O3	19.50	72.04	-0.01	664.6602	664.6593	646.6523, 300.2867, 282.2787, 264.2681, 252.2657, 60.0435
199	Cer (d18:1/25:1)	C43 H83 N O3	18.60	63.55	-3.53	662.6446	662.6426	644.6228, 300.2881, 282.2784, 264.2683, 252.2673, 60.0444
**200**	**Cer (d18:1/25:2)**	**C43 H81 N O3**	**18.41**	**61.26**	**1.29**	**660.6289**	**660.6258**	**300.2914, 282.2794, 264.2686, 252.2697, 60.0445**
**201**	**Cer (d18:1/25:4)**	**C43 H77 N O3**	**13.64**	**70.95**	**5.11**	**656.5976**	**656.5949**	**638.5964, 620.5934, 300.2920, 282.2783, 264.2673, 252.2687, 60.0437**
202	Cer (d18:1/26:0)	C44 H87 N O3	20.40	77.01	1.09	678.6759	678.6747	660.6770, 300.2862, 282.2806, 264.2682, 252.2684, 60.0444
203	Cer (d18:1/26:1)	C44 H85 N O3	19.23	69.78	2.34	676.6602	676.6574	658.6508, 640.6382, 282.2785, 264.2684, 60.0483
**204**	**Cer (d18:1/26:4)**	**C44 H77 N O3**	**14.35**	**94.37**	**2.07**	**668.5976**	**668.5991**	**650.5900, 300.2882, 282.2813, 264.2679, 60.0437**
**205**	**Cer (d18:1/26:4) isomer**	**C44 H77 N O3**	**13.20**	**77.90**	**0.99**	**668.5976**	**668.5989**	**650.5833, 632.5784, 300.2890, 282.2784, 264.2678, 252.2683, 60.0438**
**206**	**Cer (d18:1/27:5)**	**C45 H79 N O3**	**15.10**	**70.79**	**-5.50**	**682.6133**	**682.6174**	**664.6041, 646.5940, 300.2891, 282.2779, 264.2679, 252.2679, 60.0438**
207	Cer (d18:1/28:4)	C46 H83 N O3	15.40	70.34	-4.32	698.6446	698.6412	300.2862, 282.2787, 264.2677, 60.0439
**208**	**Cer (d18:1/28:5)**	**C46 H81 N O3**	**15.60**	**71.59**	**-3.93**	**696.6289**	**696.6324**	**678.6159, 660.6082, 300.2740, 282.2785, 264.2679, 252.2684, 60.0438**
**209**	**Cer (d18:1/29:5)**	**C47 H83 N O3**	**16.20**	**77.15**	**-3.69**	**710.6446**	**710.6476**	**692.6337, 674.6262, 300.2887, 282.2789, 264.2679, 252.2682, 60.0439**

**210**	**Cer (d18:1/32:4)**	**C50 H91 N O3**	**17.70**	**61.11**	**-1.99**	**754.7072**	**754.7036**	**736.6524, 718.6279, 282.2788, 264.2689, 60.0436**
211	Cer (d18:1/32:5)	C50 H89 N O3	17.65	76.56	-2.45	752.6915	752.6944	734.6812, 716.6725, 300.2906, 282.2783, 264.2678, 252.2644, 60.0437
212	Cer (d19:1/24:0)	C43 H85 N O3	19.58	76.42	1.15	664.6602	664.6588	646.6520, 296.3310, 278.2849, 266.2833, 60.0436
213	Cer (d19:2/16:0)	C35 H67 N O3	13.03	98.78	-0.26	550.5194	550.5194	532.5097, 514.4927, 502.4931, 312.2890, 294.2785, 276.2685, 264.2688, 60.0445
**214**	**Cer (d19:2/16:1)**	**C35 H65 N O3**	**12.93**	**96.12**	**-0.77**	**548.5037**	**548.5042**	**312.2895, 294.2790, 276.2687, 264.2683, 60.0444**
**215**	**Cer (d19:2/16:2)**	**C35 H63 N O3**	**10.70**	**78.16**	**-5.54**	**546.4881**	**546.4856**	**528.4767, 510.4620, 498.4630, 294.2815, 276.2684, 264.2674, 60.0445**
216	Cer (d19:2/18:2)	C37 H67 N O3	13.60	60.45	-5.37	574.5194	574.5164	294.2772, 276.2690, 264.2683
**217**	**Cer (d19:2/18:3)**	**C37 H65 N O3**	**13.42**	**72.89**	**2.89**	**572.5037**	**572.5013**	**554.4765, 312.2939, 294.2777, 276.2684, 264.2683, 60.0444**
**218**	**Cer (d19:2/18:4)**	**C37 H63 N O3**	**12.23**	**63.95**	**6.10**	**570.4881**	**570.4846**	**552.4873, 534.4846, 312.2876, 294.2788, 276.2674, 264.2675, 60.0438**
219	Cer (d19:2/20:0)	C39 H75 N O3	15.83	70.56	-3.24	606.5820	606.5815	588.5678, 570.5591, 294.2788, 276.2676, 264.2673, 60.0438
**220**	**Cer (d19:2/20:4)**	**C39 H67 N O5**	**13.35**	**90.84**	**3.17**	**598.5194**	**598.5176**	**580.5193, 562.5156, 312.3244, 294.2816, 276.2678, 264.2680, 60.0439**
**221**	**Cer (d19:2/21:5)**	**C40 H67 N O3**	**12.36**	**71.01**	**-6.14**	**610.5194**	**610.5235**	**592.5112, 574.5010, 294.2754, 276.2675, 264.2670, 60.0438**
**222**	**Cer (d19:2/22:1)**	**C41 H77 N O3**	**16.83**	**60.44**	**-0.83**	**632.5976**	**632.5950**	**614.5356, 294.2783, 276.2680, 264.2694, 60.0443**
**223**	**Cer (d19:2/24:1)**	**C43 H81 N O3**	**17.85**	**66.77**	**-2.57**	**660.6267**	**660.6268**	**642.6215, 624.5982, 612.6075, 294.2784, 276.2682, 264.2677, 60.0444**
**224**	**Cer (d19:2/24:2)**	**C43 H79 N O3**	**18.00**	**61.37**	**2.94**	**658.6133**	**658.6100**	**640.6160, 294.2813, 276.2669, 264.2683, 60.0438**
**225**	**Cer (d19:3/17:4)**	**C36 H59 N O3**	**11.28**	**70.54**	**2.13**	**554.4568**	**554.4541**	**536.4232, 292.2621, 274.2524, 262.2497, 60.0438**
226	Cer (d20:1/24:1)	C44 H85 N O3	19.54	80.43	-2.35	676.6602	676.6584	658.6530, 310.3071, 292.2995, 280.3015, 60.0438
227	Cer (d20:1/25:0)	C45 H89 N O3	21.30	65.70	-1.52	692.6915	692.6894	310.3110, 292.2997, 280.2976, 60.0443
228	Cer (d20:1/26:0)	C46 H91 N O3	22.00	63.42	1.16	706.7072	706.7055	688.7034, 670.6835, 328.3253, 310.3105, 292.2999, 280.2994, 60.0444
**229**	**Cer (d20:1/26:2)**	**C46 H87 N O3**	**21.17**	**60.14**	**-3.86**	**702.6759**	**702.6720**	**684.6607, 328.3002, 310.3127, 292.2998, 280.2994, 60.0445**
230	Cer (d20:1/27:0)	C47 H93 N O3	22.60	64.89	-0.93	720.7228	720.7207	702.7083, 684.7016, 310.3095, 292.2998, 280.3012, 60.0444
**231**	**Cer (d20:2/28:0)**	**C48 H93 N O3**	**21.24**	**83.22**	**-4.72**	**732.7228**	**732.7198**	**714.7022, 326.3055, 308.2946, 290.2848, 278.2872, 60.0445**

**232**	**Cer (d21:1/28:0)**	**C49 H97 N O3**	**23.42**	**68.25**	**-1.47**	**748.7541**	**748.7518**	**730.6799, 324.3259, 306.3160, 294.3166, 60.0442**
**233**	**Cer (d22:1/26:2)**	**C48 H91 N O3**	**22.42**	**74.07**	**-4.33**	**730.7072**	**730.7034**	**712.7041, 356.3303, 338.3414, 320.3307, 308.3314, 60.0442**
234	Cer (d22:1/26:0)	C48 H95 N O3	22.99	62.45	1.73	734.7385	734.7370	716.7297, 698.7081, 338.3418, 320.3309, 308.3295, 60.0445
**235**	**Cer (d22:1/27:2)**	**C49 H93 N O3**	**22.89**	**71.79**	**-5.40**	**744.7228**	**744.7184**	**726.6413, 320.3312, 308.3324, 60.0445**
236	Cer (d22:1/28:0)	C50 H99 N O3	23.85	78.70	-0.61	762.7698	762.7679	744.7567, 726.7396, 338.3407, 320.3318, 308.3309, 60.0444
**237**	**Cer (d14:1/18:3(OH))**	**C32 H57 N O4**	**13.62**	**97.64**	**-0.54**	**520.4360**	**520.4354**	**226.2176, 208.2065, 196.2062, 60.0445**
**238**	**Cer (d14:2/14:3(OH))**	**C28 H47 N O4**	**9.03**	**89.60**	**-1.61**	**462.3578**	**462.3571**	**444.3234, 206.1909, 60.0432**
239	Cer (d18:1/24:0(OH))	C42 H83 N O4	17.98	86.98	0.55	666.6395	666.6397	648.6270, 630.6195, 300.2887, 282.2787, 264.2681, 252.2683, 60.0439
**240**	**Cer (d18:1/26:2(OH))**	**C44 H83 N O4**	**18.55**	**87.88**	**-3.14**	**690.6395**	**690.6376**	**672.6232, 300.2876, 282.2766, 264.2685, 252.2686, 60.0444**
241	Cer (d19:2/16:1(OH))	C35 H65 N O4	11.98	99.09	-0.05	564.4986	564.4986	546.4859, 528.4769, 510.4669, 498.4673, 312.2901, 294.2786, 276.2685, 264.2686, 60.0445
**242**	**Cer (d19:2/25:2(OH))**	**C44 H81 N O4**	**17.90**	**68.92**	**-0.54**	**688.6238**	**688.6235**	**670.6204, 294.2822, 276.2681, 264.2685, 60.0444**
**243**	**Cer (d19:3/16:1(OH))**	**C35 H63 N O4**	**10.27**	**86.35**	**-1.54**	**562.4830**	**562.4823**	**544.4573, 526.4616, 514.4674, 310.2741, 292.2638, 274.2525, 262.2538, 60.0443**
**244**	**Cer (d20:1/26:2(OH))**	**C46 H87 N O4**	**19.50**	**74.11**	**-5.66**	**718.6708**	**718.6666**	**700.6493, 328.3003, 310.2958, 292.2999, 280.3026, 60.0442**
**245**	**Cer (d20:1/28:2(OH))**	**C48 H91 N O4**	**21.22**	**81.74**	**-4.48**	**746.7021**	**746.6990**	**728.6892, 328.3194, 310.3141, 292.3000, 280.3083, 60.0444**
246	Cer (t18:1/16:0)	C34 H67 N O4	12.07	98.07	-0.36	554.5143	554.5140	536.5034, 518.4917, 500.4812, 488.4821, 316.2846, 298.2742, 280.2636, 262.2535, 250.2534, 60.0445
**247**	**Cer (t18:1/16:1)**	**C34 H65 N O4**	**11.36**	**80.67**	**0.67**	**552.4986**	**552.4988**	**534.4856, 516.4770, 316.2805, 298.2729, 280.2632, 262.2532, 250.2522, 60.0438**
**248**	**Cer (t18:1/17:0)**	**C35 H69 N O4**	**12.61**	**99.87**	**0.34**	**568.5299**	**568.5297**	**550.5129, 532.5038, 514.4871, 502.4912, 316.2853, 298.2738, 280.2637, 262.2534, 250.2529, 60.0444**

249	Cer (t18:1/18:0)	C36 H71 N O4	12.90	99.97	-0.14	582.5456	582.5457	564.5339, 546.5239, 528.5136, 316.2842, 298.2728, 280.2628, 262.2524, 250.2521, 60.0439
**250**	**Cer (t18:1/18:1)**	**C36 H69 N O4**	**12.36**	**98.28**	**-0.95**	**580.5299**	**580.5303**	**562.5187, 544.5075, 526.4959, 316.2841, 298.2731, 280.2626, 262.2523, 250.2522, 60.0439**
**251**	**Cer (t18:1/18:2) isomer**	**C36 H67 N O4**	**11.68**	**98.06**	**-0.26**	**578.5143**	**578.5140**	**560.4973, 542.4909, 524.4842, 512.4799, 316.2846, 298.2739, 280.2634, 262.2533, 250.2535, 60.0445**
**252**	**Cer (t18:1/18:2)**	**C36 H67 N O4**	**12.50**	**70.64**	**2.72**	**578.5143**	**578.5128**	**560.4902, 542.4856, 524.4864, 316.2840, 298.2732, 280.2627, 262.2526, 250.2529, 60.0439**
**253**	**Cer (t18:1/18:3)**	**C36 H65 N O4**	**12.10**	**96.31**	**1.76**	**576.4986**	**576.4976**	**558.4670, 522.4742, 316.2826, 298.2727, 280.2623, 262.2526, 250.2160, 60.0438**
**254**	**Cer (t18:1/18:4)**	**C36 H63 N O4**	**12.25**	**84.80**	**-2.39**	**574.4830**	**574.4811**	**556.4733, 538.4963, 526.4603, 316.2598, 298.2548, 280.2441, 262.2545, 250.2498, 60.0444**
**255**	**Cer (t18:1/18:5)**	**C36 H61 N O4**	**11.65**	**71.61**	**-2.02**	**572.4673**	**572.4655**	**316.2662, 298.2726, 280.2635, 262.2536, 250.2538, 60.0444**
**256**	**Cer (t18:1/19:0)**	**C37 H73 N O4**	**13.97**	**72.22**	**1.09**	**596.5612**	**596.5609**	**578.5503, 560.5393, 316.2833, 298.2719, 280.2616, 262.2507, 250.2536, 60.0438**
257	Cer (t18:1/20:0)	C38 H75 N O4	14.53	89.85	2.91	610.5769	610.5751	592.5653, 574.5535, 556.5479, 316.2853, 298.2732, 280.2626, 262.2534, 250.2535, 60.0439
**258**	**Cer (t18:1/20:3)**	**C38 H69 N O4**	**13.75**	**72.92**	**-0.03**	**604.5299**	**604.5302**	**586.5114, 298.2549, 280.2431, 262.2524, 250.1978, 60.0438**
259	Cer (t18:1/21:0)	C39 H77 N O4	15.18	67.91	2.25	624.5925	624.5937	606.5834, 588.5681, 570.5621, 316.2832, 298.2738, 280.2632, 262.2539, 250.2529, 60.0445
260	Cer (t18:1/22:0)	C40 H79 N O4	15.85	97.62	0.17	638.6082	638.6082	620.5952, 602.5859, 584.5758, 316.2848, 298.2741, 280.2635, 262.2532, 250.2532, 60.0445
**261**	**Cer (t18:1/22:2)**	**C40 H75 N O4**	**15.06**	**60.13**	**4.36**	**634.5769**	**634.5737**	**616.5468, 598.5342, 316.2847, 298.2728, 280.2610, 262.2511,**

								**250.2529, 60.0438**
**262**	**Cer (t18:1/22:3)**	**C40 H73 N O4**	**15.20**	**63.73**	**-1.93**	**632.5612**	**632.5615**	**316.2800, 298.2719, 280.2616, 262.2519, 250.2531, 60.0438**
**263**	**Cer (t18:1/22:4)**	**C40 H71 N O4**	**14.78**	**83.30**	**-1.66**	**630.5456**	**630.5442**	**612.5304, 582.5223, 298.3091, 280.2625, 262.2531, 250.2537, 60.0445**
264	Cer (t18:1/23:0)	C41 H81 N O4	16.50	92.67	0.77	652.6238	652.6242	634.6119, 616.6029, 598.5842, 316.2845, 298.2736, 280.2635, 262.2533, 250.2528, 60.0445
265	Cer (t18:1/24:0)	C42 H83 N O4	17.22	99.65	0.15	666.6395	666.6397	648.6274, 630.6160, 612.6058, 600.6070, 316.2843, 298.2740, 280.2634, 262.2533, 250.2533, 60.0445
266	Cer (t18:1/24:1)	C42 H81 N O4	16.02	69.30	-5.37	664.6238	664.6203	646.6081, 628.6009, 610.5899, 316.2840, 298.2740, 280.2635, 262.2534, 250.2534, 60.0445
**267**	**Cer (t18:1/24:2)**	**C42 H79 N O4**	**16.38**	**85.64**	**1.95**	**662.6082**	**662.6066**	**644.5780, 626.5889, 316.2825, 298.2740, 280.2626, 262.2524, 250.2533, 60.0443**
268	Cer (t18:1/25:0)	C43 H85 N O4	17.92	89.09	-1.61	680.6551	680.6538	662.6423, 644.6316, 626.6167, 316.2841, 298.2737, 280.2636, 262.2533, 250.2534, 60.0444
269	Cer (t18:1/25:1)	C43 H83 N O4	16.75	70.78	1,09	678.6395	678.6358	660.6205, 642.6185, 316.2843, 298.2730, 280.2629, 262.2528, 250.2533, 60.0439
**270**	**Cer (t18:1/25:2)**	**C43 H81 N O4**	**17.03**	**62.43**	**-0.21**	**676.6238**	**676.6210**	**658.6035, 316.2874, 298.2738, 280.2636, 262.252, 250.2522, 60.0444**
**271**	**Cer (t18:1/28:2)**	**C46 H87 N O4**	**18.82**	**74.21**	**-5.47**	**718.6708**	**718.6671**	**700.6501, 316.3009, 298.2650, 280.2623, 262.2535, 250.2530, 60.0446**
**272**	**Cer (t19:1/18:2)**	**C37 H69 N O4**	**13.13**	**60.05**	**0.69**	**592.5299**	**592.5319**	**574.5200, 556.5060, 538.4960, 330.3001, 312.2902, 294.2776, 276.2682, 264.2690, 60.0444**
**273**	**Cer (t19:1/18:4)**	**C37 H65 N O4**	**12.43**	**69.84**	**4.72**	**588.4986**	**588.5018**	**570.4809, 552.5144, 540.4658, 330.2678, 312.2711, 294.2750, 276.2686, 264.2661, 60.0444**
**274**	**Cer (t19:1/24:3)**	**C43 H79 N O4**	**16.48**	**63.51**	**0.95**	**674.6082**	**674.6109**	**656.5833, 638.6259, 312.2902, 294.2796, 276.2683, 264.2689, 60.0444**
**275**	**Cer (t18:1/14:0(OH))**	**C32 H63 N O5**	**10.60**	**84.32**	**-4.34**	**542.4779**	**542.4758**	**524.4441, 506.4253, 316.2635, 298.2735, 280.2637, 262.2541,**

								**250.2532, 60.0444**
276	Cer (t18:1/16:0(OH))	C34 H67 N O5	11.65	98.64	-0.48	570.5092	570.5090	552.5002, 534.4891, 516.4795, 498.4646, 316.2839, 298.2745, 280.2638, 262.2539, 250.2532, 60.0446
**277**	**Cer (t18:1/17:1(OH))**	**C35 H67 N O5**	**10.88**	**69.44**	**2.17**	**582.5092**	**582.5083**	**564.4905, 546.4905, 528.4662, 510.4205, 316.2708, 298.2720, 280.2633, 262.2536, 250.2218, 60.0438**
278	Cer (t18:1/18:0(OH))	C36 H61 N O5	12.78	93.94	0.01	598.5405	598.5408	580.5296, 562.5184, 544.5077, 526.4997, 316.2731, 298.2731, 280.2628, 262.2527, 250.2529, 60.0439
**279**	**Cer (t18:1/18:1(OH))**	**C36 H69 N O5**	**11.80**	**80.41**	**1.31**	**596.5249**	**596.5253**	**578.5137, 560.5027, 542.4910, 524.4786, 316.2838, 298.2731, 280.2626, 262.2529, 250.2523, 60.0439**
**280**	**Cer (t18:1/18:2(OH))**	**C36 H67 N O5**	**11.31**	**76.49**	**-0.25**	**594.5092**	**594.5089**	**576.4935, 558.4820, 316.2872, 298.2740, 280.2632, 262.2531, 250.2535, 60.0444**
281	Cer (t18:1/20:0(OH))	C38 H75 N O5	13.94	98.20	-0.72	626.5718	626.5714	608.5597, 590.5487, 572.5378, 554.5298, 316.2840, 298.2738, 280.2635, 262.2532, 250.2534, 60.0444
**282**	**Cer (t18:1/20:1(OH))**	**C38 H73 N O5**	**12.91**	**81.77**	**3.62**	**624.5562**	**624.5544**	**606.5258, 588.5149, 570.5020, 558.4867, 316.2678, 298.2537, 280.2629, 262.2524, 250.2495, 60.0439**
283	Cer (t18:1/21:0(OH))	C39 H77 N O5	14.54	95.50	-1.21	640.5875	640.5867	622.5744, 604.5642, 586.5536, 568.5433, 316.2847, 298.2737, 280.2631, 262.2534, 250.2532, 60.0444
284	Cer (t18:1/22:0(OH))	C40 H79 N O5	15.20	99.54	-0.08	654.6031	654.6029	636.5919, 618.5810, 600.5713, 582.5578, 316.2846, 298.2743, 280.2639, 262.2537, 250.2534, 60.0446
285	Cer (t18:1/22:1(OH))	C40 H77 N O5	14.18	75.26	-3.01	652.5875	652.5872	634.5772, 616.5650, 598.5531, 580.5426, 316.2847, 298.2741, 280.2636, 262.2534, 250.2533, 60.0445
**286**	**Cer (t18:1/22:2(OH))**	**C40 H75 N O5**	**13.31**	**79.69**	**2.01**	**650.5718**	**650.5719**	**632.5596, 614.5493, 596.5443, 578.5311, 316.2829, 298.2727, 280.2643, 262.2515, 250.2188, 60.0438**

287	Cer (t18:1/23:0(OH))	C41 H81 N O5	15.90	98.18	0.04	668.6188	668.6186	650.6073, 632.5966, 614.5859, 596.5749, 316.2842, 298.2743, 280.2638, 262.2535, 250.2532, 60.0446
288	Cer (t18:1/23:1(OH))	C41 H79 N O5	14.85	99.57	0.34	666.6031	666.6034	648.5920, 630.5808, 612.5697, 600.5321, 594.5572, 316.2837, 298.2740, 280.2635, 262.2534, 250.253, 60.0445
289	Cer (t18:1/24:0(OH))	C42 H83 N O5	16.43	98.79	-0.04	682.6344	682.6344	664.6237, 646.6129, 628.6019, 610.5907, 316.2845, 298.2748, 280.2642, 262.2537, 250.2535, 60.0445
290	Cer (t18:1/24:1(OH))	C42 H81 N O5	15.50	75.04	-6.75	680.6188	680.6139	662.6078, 644.5969, 626.5858, 608.5744, 316.2844, 298.2744, 280.2640, 262.2538, 250.2533, 60.0446
**291**	**Cer (t18:1/24:2(OH))**	**C42 H79 N O5**	**15.76**	**70.31**	**-1.28**	**678.6031**	**678.6025**	**660.5742, 642.5699, 316.2881, 298.2753, 280.2623, 262.2536, 250.2531, 60.0445**
292	Cer (t18:1/25:0(OH))	C43 H85 N O5	17.20	99.74	-0.16	696.6501	696.6498	678.6384, 660.6277, 642.6169, 624.6067, 612.6059, 316.2839, 298.2742, 280.2637, 262.2535, 250.2531, 60.0445
293	Cer (t18:1/25:1(OH))	C43 H83 N O5	16.20	90.26	-3.91	694.6344	694.6318	676.6216, 658.6117, 640.6023, 622.5935, 610.5932, 316.2849, 298.2743, 280.2636, 262.2536, 250.2534, 60.0445
**294**	**Cer (t18:1/25:2(OH))**	**C43 H81 N O5**	**16.41**	**79.78**	**0.90**	**692.6188**	**692.6172**	**674.6149, 316.2841, 298.2736, 280.2631, 262.2531, 250.2528, 60.0444**
295	Cer (t18:1/26:0(OH))	C44 H87 N O5	17.97	94.21	0.28	710.6657	710.6655	692.6539, 674.6427, 656.6334, 638.6195, 316.2841, 298.2740, 280.2634, 262.2532, 250.2534, 60.0445
296	Cer (t18:1/24:0(OH)) isomer	C42 H83 N O5	16.70	91.89	-3.09	682.6344	682.6363	316.2832, 298.2739, 280.2632, 262.2535, 250.2535, 60.0445
**297**	**Cer (t19:1/16:0(OH))**	**C35 H69 N O5**	**12.38**	**73.27**	**-2.15**	**584.5249**	**584.5251**	**566.5127, 548.5035, 530.4901, 512.4839, 330.3000, 312.2896, 294.2784, 276.2682, 264.2679, 60.0444**
**298**	**Cer (t19:1/18:0(OH))**	**C37 H73 N O5**	**13.62**	**98.14**	**-0.08**	**612.5562**	**612.5563**	**594.5453, 576.5334, 558.5239, 540.5070, 330.2995, 312.2898, 294.2788, 276.2685, 264.2682, 60.0444**
**299**	**Cer (t14:1/25:5(tOH))**	**C39 H67 N O7**	**13.47**	**96.18**	**1.13**	**662.4990**	**662.5003**	**644.4842, 206.1907, 194.1889, 60.0444**

**300**	**Cer (t14:1/22:1(tOH))**	**C36 H69 N O7**	**13.52**	**93.45**	**-0.88**	**628.5147**	**628.5139**	**610.5070, 592.4891, 224.1960, 206.1904, 194.1867, 60.0446**
**301**	**Cer (t18:1/23:5(tOH))**	**C41 H71 N O7**	**15.62**	**96.85**	**1.61**	**690.5303**	**690.5315**	**672.5053, 654.5093, 298.2734, 280.2632, 262.2542, 250.2532, 60.0445**
**302**	**Cer (t20:0/33:5(tOH))**	**C53 H95 N O7**	**20.40**	**84.46**	**-0.20**	**858.7181**	**858.7198**	**840.6983, 328.3243, 310.3140, 292.3008, 280.2970, 60.0444**
**303**	**Cer (d16:0/35:1(dOH))**	**C51 H101 N O5**	**17.57**	**88.25**	**-2.28**	**808.7753**	**808.7726**	**790.7624, 772.7514, 754.7339, 742.6503, 292.3002, 274.2732, 256.2636, 60.0444**
**304**	**Cer (t14:1/16:1(dOH))**	**C30 H57 N O6**	**10.45**	**88.75**	**-4.41**	**528.4259**	**528.4237**	**510.4143, 492.4048, 224.2005, 206.1907, 194.1899, 60.0443**
**305**	**Cer (t14:1/16:0(dOH))**	**C30 H59 N O6**	**10.52**	**78.15**	**-7.13**	**530.4415**	**530.4378**	**512.4034, 242.2020, 224.2008, 206.1905, 194.1910, 60.0444**
**306**	**Cer (t14:1/22:1(dOH))**	**C36 H69 N O6**	**14.23**	**89.45**	**-2.04**	**612.5198**	**612.5184**	**594.4955, 206.1907, 60.0444**
**307**	**Cer (t14:1/24:3(dOH))**	**C38 H69 N O6**	**12.25**	**79.78**	**4.05**	**636.5198**	**636.5215**	**618.5123, 600.4934, 224.1984, 206.1905, 194.1917, 60.0445**
**308**	**Cer (t14:1/25:3(dOH))**	**C39 H71 N O6**	**12.40**	**85.88**	**4.23**	**650.5354**	**650.5378**	**632.4836, 614.5106, 596.4895, 206.1908, 194.1917, 60.0444**
**309**	**Cer (t14:1/26:0(dOH))**	**C40 H79 N O6**	**13.18**	**85.82**	**1.13**	**670.5980**	**670.5986**	**652.5095, 224.2007, 206.1906, 60.0442**
**310**	**Cer (t18:0/23:4(dOH))**	**C41 H75 N O6**	**12.68**	**75.74**	**-1.98**	**678.5667**	**678.5640**	**660.5306, 642.5226, 624.5197, 606.5229, 588.5120, 282.2790, 264.2688, 252.2674, 60.0446**
**311**	**Cer (t18:0/42:1(dOH))**	**C60 H117 N O6**	**25.19**	**93.66**	**-2.24**	**948.8954**	**948.8931**	**930.8809, 912.8630, 894.8682, 318.3006, 300.2897, 282.2790, 264.2683, 252.2694, 60.0445**
312	Cer (t18:1/24:0(dOH))	C42 H83 N O6	15.20	89.04	-0.56	698.6293	698.6294	680.6160, 662.6006, 644.5878, 626.5221, 298.2730, 280.2628, 262.2537, 250.2530, 60.0444
**313**	**Cer (t18:1/29:2(dOH))**	**C47 H89 N O6**	**17.30**	**84.29**	**1.08**	**764.6763**	**764.6775**	**746.6444, 728.6761, 298.2732, 280.2635, 262.2530, 250.2518, 60.0443**
**314**	**Cer (t18:1/42:1(dOH))**	**C60 H115 N O6**	**24.50**	**84.68**	**-2.25**	**946.8797**	**946.8772**	**928.8625, 910.8667, 316.2841, 298.2740, 280.2623, 262.2525, 250.2535, 60.0442**
315	Cer (d18:2/16:1)	C34 H63 N O3	12.25	97.16	0.17	534.4881	534.4880	280.2631, 262.2531, 250.2532, 60.0445
**316**	**Cer (d18:2/16:2)**	**C34 H61 N O3**	**11.65**	**71.45**	**0.24**	**532.4724**	**532.4732**	**514.4609, 496.4503, 484.4485, 298.2506, 280.2633, 262.2533, 250.2532, 60.0444**

**317**	**Cer (d18:2/16:3)**	**C34 H59 N O3**	**11.61**	**63.60**	**3.70**	**530.4568**	**530.4543**	**298.2545, 280.2631, 262.2532, 250.2530, 60.0444**
318	Cer (d18:2/18:2)	C36 H65 N O3	13.25	70.68	-3.52	560.5037	560.5033	542.4927, 524.4891, 280.2626, 262.2525, 250.2527, 60.0438
**319**	**Cer (d18:2/18:3)**	**C36 H63 N O3**	**12.82**	**88.60**	**-3.35**	**558.4881**	**558.4860**	**540.4748, 280.2629, 262.2538, 250.2534, 60.0445**
**320**	**Cer (d18:2/18:4)**	**C36 H61 N O3**	**12.00**	**80.97**	**4.51**	**556.4724**	**556.4700**	**538.4693, 298.2719, 280.2615, 262.2525, 60.0439**
**321**	**Cer (d18:2/20:4)**	**C38 H65 N O3**	**12.76**	**69.46**	**7.36**	**584.5037**	**584.4994**	**566.4892, 262.2528, 250.1914, 60.0438**
**322**	**Cer (d18:2/20:5)**	**C38 H63 N O3**	**10.80**	**70.99**	**1.35**	**582.4881**	**582.4878**	**298.2728, 280.2620, 262.2522, 250.2531, 60.0438**
**323**	**Cer (d18:2/21:6)**	**C39 H63 N O3**	**11.90**	**67.85**	**2.25**	**594.4881**	**594.4871**	**298.2737, 280.2622, 262.2519, 250.2525, 60.0438**
**324**	**Cer (d18:2/22:2)**	**C40 H73 N O3**	**16.05**	**62.40**	**0.06**	**616.5663**	**616.5636**	**598.5538, 568.5054, 298.2556, 280.2603, 262.2536, 250.2544, 60.0445**
**325**	**Cer (d18:2/22:3)**	**C40 H71 N O3**	**15.32**	**80.34**	**-4.22**	**614.5507**	**614.5483**	**596.5360, 566.5336, 280.2640, 262.2526, 250.2562, 60.0443**
**326**	**Cer (d18:2/22:5)**	**C40 H67 N O3**	**11.80**	**81.34**	**-3.89**	**610.5194**	**610.522**	**592.5096, 574.4995, 298.2544, 280.2623, 262.2520, 250.2536, 60.0438**
**327**	**Cer (d18:2/23:2)**	**C41 H75 N O3**	**16.64**	**70.34**	**1.23**	**630.5820**	**630.5814**	**298.2717, 280.2628, 262.2519, 250.2534, 60.0437**
**328**	**Cer (d18:2/23:3)**	**C41 H73 N O3**	**16.10**	**80.09**	**4.40**	**628.5663**	**628.5636**	**610.5100, 280.2624, 262.2528, 250.2525, 60.0444**
**329**	**Cer (d18:2/23:5)**	**C41 H69 N O3**	**12.38**	**76.57**	**-4.00**	**624.5350**	**624.5379**	**606.5288, 588.5229, 280.2615, 262.2545, 250.2152, 60.4380**
**330**	**Cer (d18:2/23:5) isomer**	**C41 H69 N O3**	**12.68**	**76.00**	**-4.83**	**624.5350**	**624.5385**	**606.5261, 588.5153, 576.5145, 298.2692, 280.2668, 262.2523, 250.2538, 60.0438**
**331**	**Cer (d18:2/23:6)**	**C41 H67 N O3**	**11.88**	**74.18**	**-5.81**	**622.5194**	**622.5232**	**604.5097, 586.4975, 298.2719, 280.2618, 262.2653, 250.2506, 60.0438**
332	Cer (d18:2/24:2)	C42 H77 N O3	17.28	62.12	0.47	644.5976	644.5946	626.5802, 298.2727, 280.2618, 262.2527, 250.2538, 60.0445
**333**	**Cer (d18:2/24:3)**	**C42 H75 N O3**	**16.76**	**71.64**	**4.65**	**642.5820**	**642.5793**	**624.5718, 606.6093, 280.2631, 262.2526, 250.2534, 60.0441**
**334**	**Cer (d18:2/24:5)**	**C42 H71 N O3**	**12.90**	**75.36**	**-3.74**	**638.5507**	**638.5537**	**620.5418, 602.5312, 298.2736, 280.2616, 262.2525, 250.2528, 60.0439**
**335**	**Cer (d18:2/25:4)**	**C43 H75 N O3**	**14.35**	**71.55**	**-0.76**	**654.5820**	**654.5842**	**636.5842, 618.5721, 298.2722, 280.2618, 262.2542, 60.0438**
**336**	**Cer (d18:2/25:5)**	**C43 H73 N O3**	**13.50**	**66.86**	**-3.43**	**652.5663**	**652.5705**	**634.5584, 616.5488, 280.2664, 262.2519, 250.2529, 60.0438**
**337**	**Cer (d18:2/26:1)**	**C44 H83 N O3**	**19.29**	**81.79**	**3.66**	**674.6446**	**674.6417**	**280.2630, 262.2520, 250.2538, 60.0444**
338	Cer (d18:2/26:4) isomer	C44 H75 N O3	12.65	84.76	-2.07	666.5820	666.5839	648.5699, 630.6019, 298.2825, 280.2615, 262.2528

339	Cer (d18:2/26:4)	C44 H75 N O3	14.65	84.28	-3.52	666.5820	666.5842	648.5761, 630.5651, 298.2724, 280.2613, 262.2519, 60.0437
**340**	**Cer (d18:2/27:5)**	**C45 H77 N O3**	**14.60**	**67.44**	**-6.98**	**680.5976**	**680.6025**	**662.5883, 644.5798, 298.2722, 280.2623, 262.2522, 250.2533, 60.0438**
**341**	**Cer (d18:2/27:6)**	**C45 H75 N O3**	**13.80**	**66.51**	**-5.37**	**678.5820**	**678.5849**	**660.5730, 642.5627, 298.2722, 280.2631, 262.2530, 250.2154, 60.0439**
**342**	**Cer (d18:2/28:5)**	**C46 H79 N O3**	**15.10**	**71.25**	**-4.67**	**694.6133**	**694.6169**	**676.6053, 658.5963, 298.2730, 280.2623, 262.2525, 250.2523, 60.0438**
**343**	**Cer (d18:2/29:5)**	**C47 H81 N O3**	**15.65**	**75.84**	**-4.36**	**708.6289**	**708.6324**	**690.6200, 672.6090, 298.2757, 280.2621, 262.2526, 250.2538, 60.0438**
**344**	**Cer (d18:2/29:6)**	**C47 H79 N O3**	**14.80**	**75.21**	**-5.34**	**706.6133**	**706.6174**	**688.6041, 670.5932, 298.2761, 280.2622, 262.2523, 250.2517, 60.0438**
**345**	**Cer (d18:2/32:5)**	**C50 H87 N O3**	**16.97**	**80.75**	**-2.99**	**750.6759**	**750.6785**	**732.6651, 714.6544, 298.2742, 280.2615, 262.2526, 60.0437**
**346**	**Cer (d18:2/32:6)**	**C50 H85 N O3**	**16.00**	**80.28**	**-2.62**	**748.6602**	**748.6627**	**730.6497, 712.6394, 298.2758, 280.2614, 262.2520, 60.0435**

*** 171 SPLs that were bolded in table were identified in this study firstly.**

Figure S1. Base peak chromatograms (BPC) of blank sample (A), sphingoid base fraction (B) and ceramide fraction (C). The extracted compound chromatograms (labeled above BPC) obtained on UHPLC-UHD-Q-TOF-MS after separation of sphingoid base and ceramide fractions by using silica gel and amino silica gel column chromatographies.

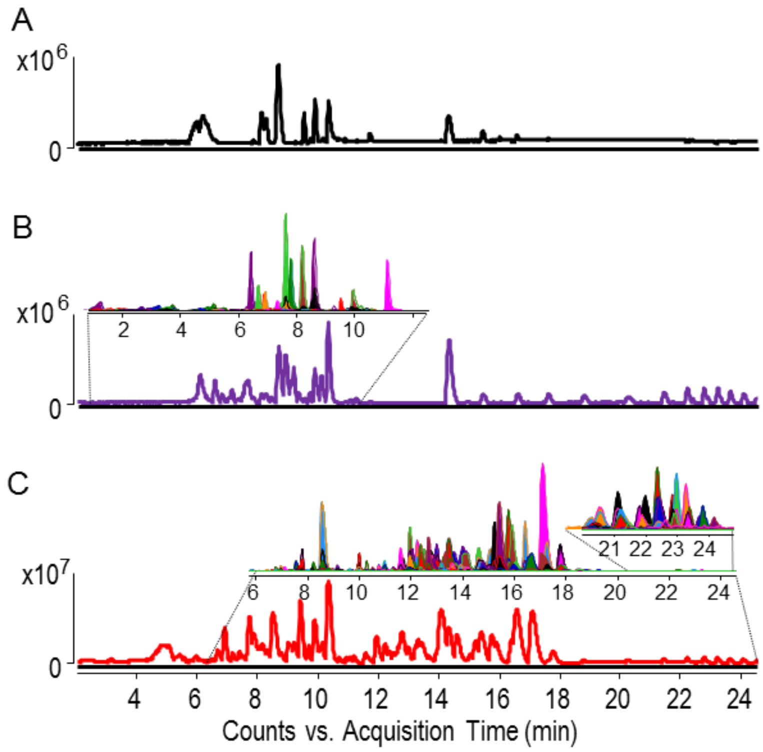

Figure S2. Comparison of extracted ion chromatographies (EICs) of representative SPLs in samples before and after silica gel and amino silica gel column chromatographies separation.

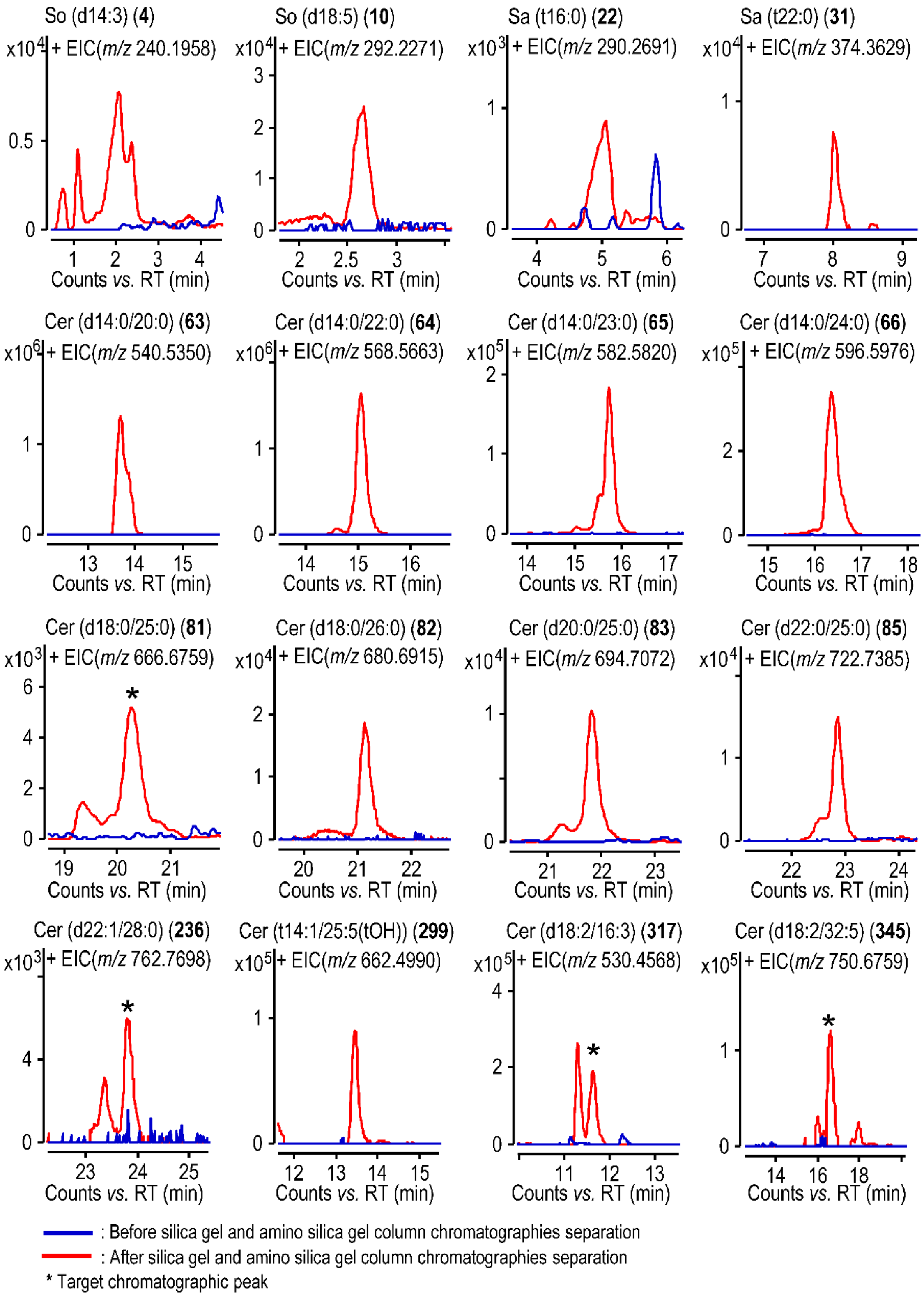

Figure S3. The *de novo* sphingolipid biosynthetic pathway.

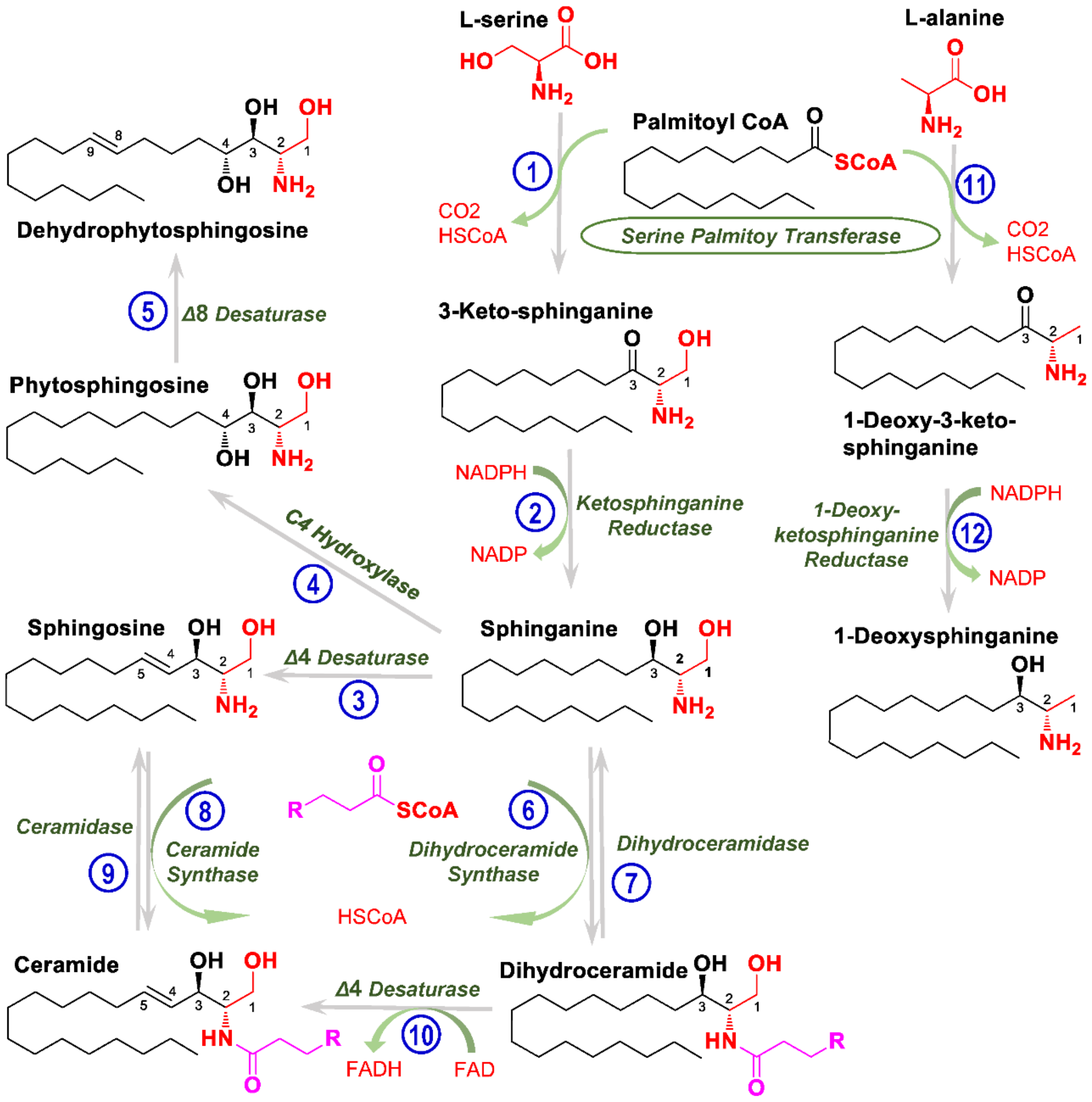

Figure S4. The synthetic reactions of 3 SPLs in this work. Hydrogenation (H_2, 10% Pd/C, MeOH) of the unsaturated SPL standards afforded saturated SPLs which were identified by UHPLC-UHD-Q-TOF-MS.

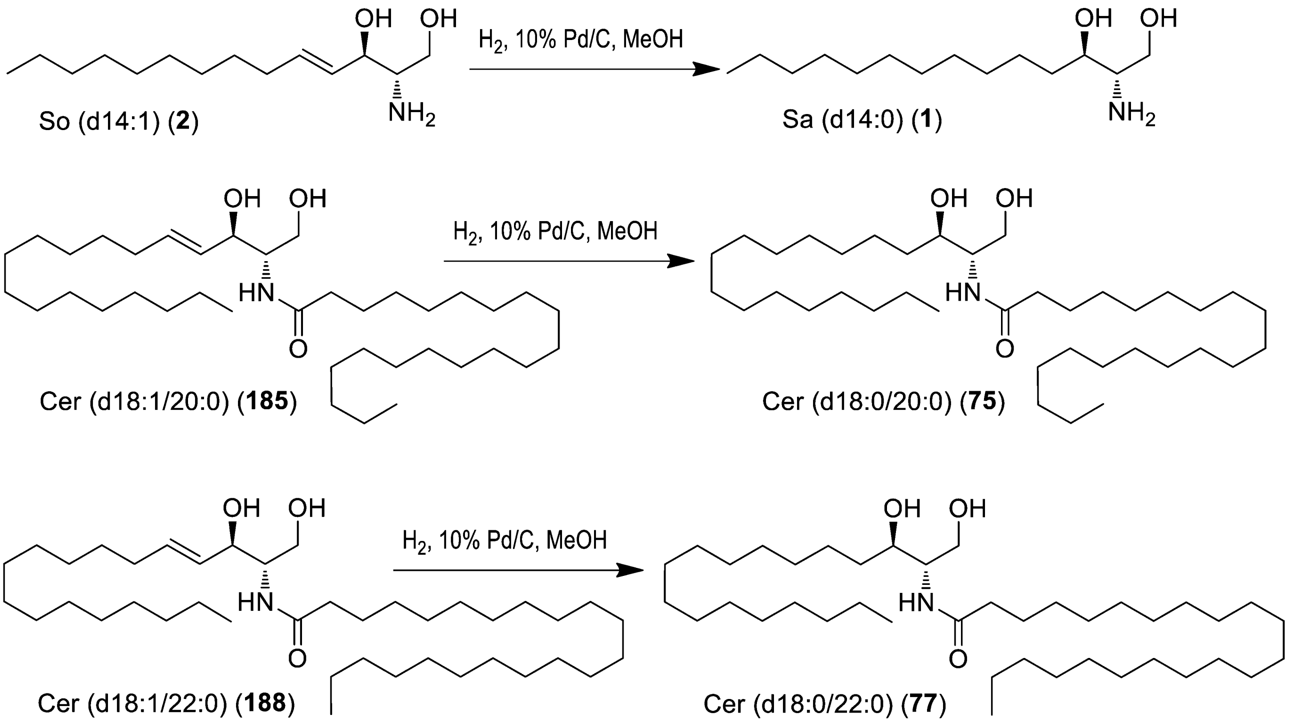

Figure S5. Comparison of the UHPLC RT values between 6 sphingoid bases and 16 ceramides in wild Cordyceps and corresponding SPL standards.

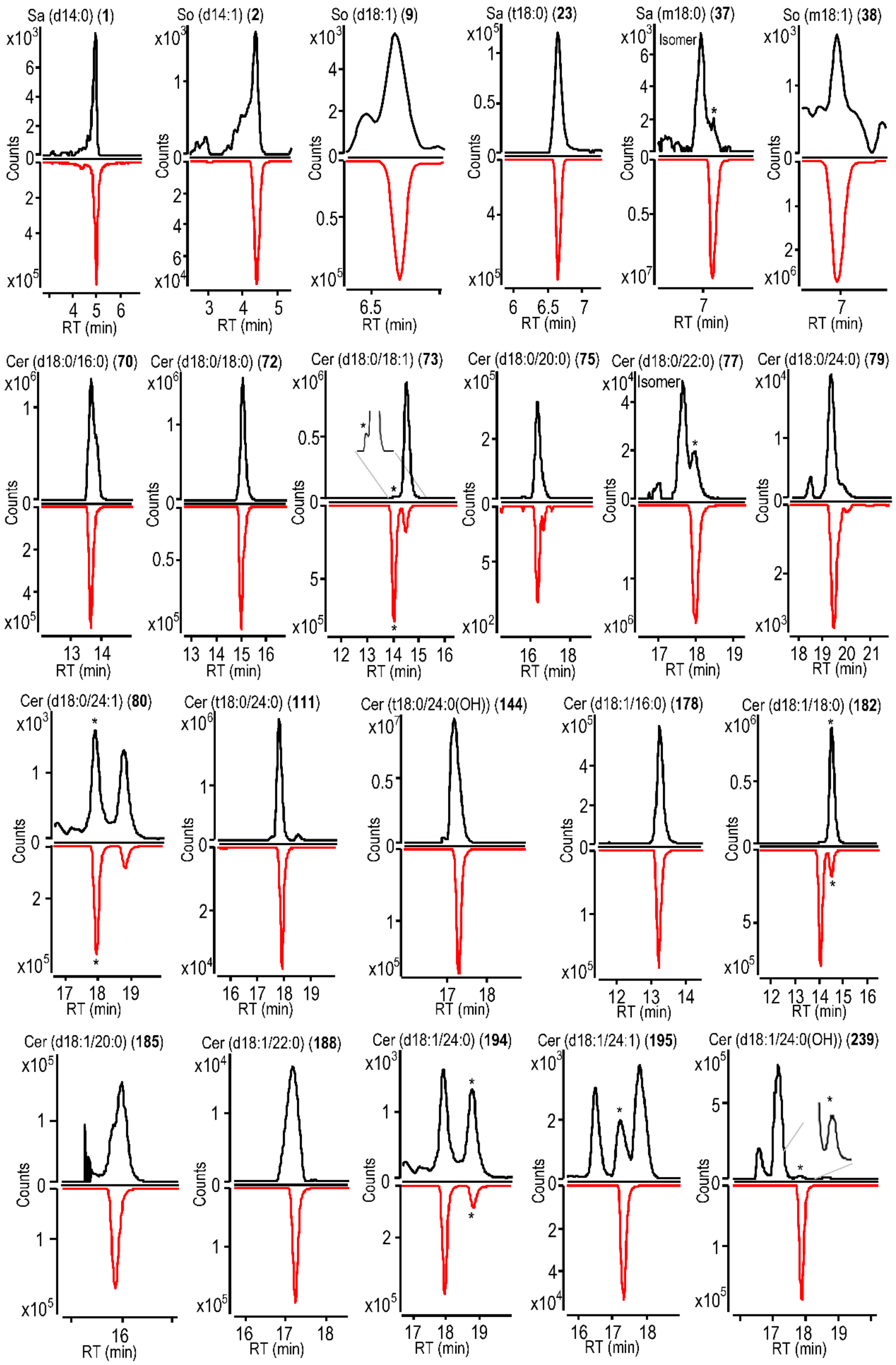

Figure S6. The chromatographic retention time rule of ceramides. X-axis stands for the number of carbon atoms in N-acyl chain of ceramides; Y-axis stands for retention time.

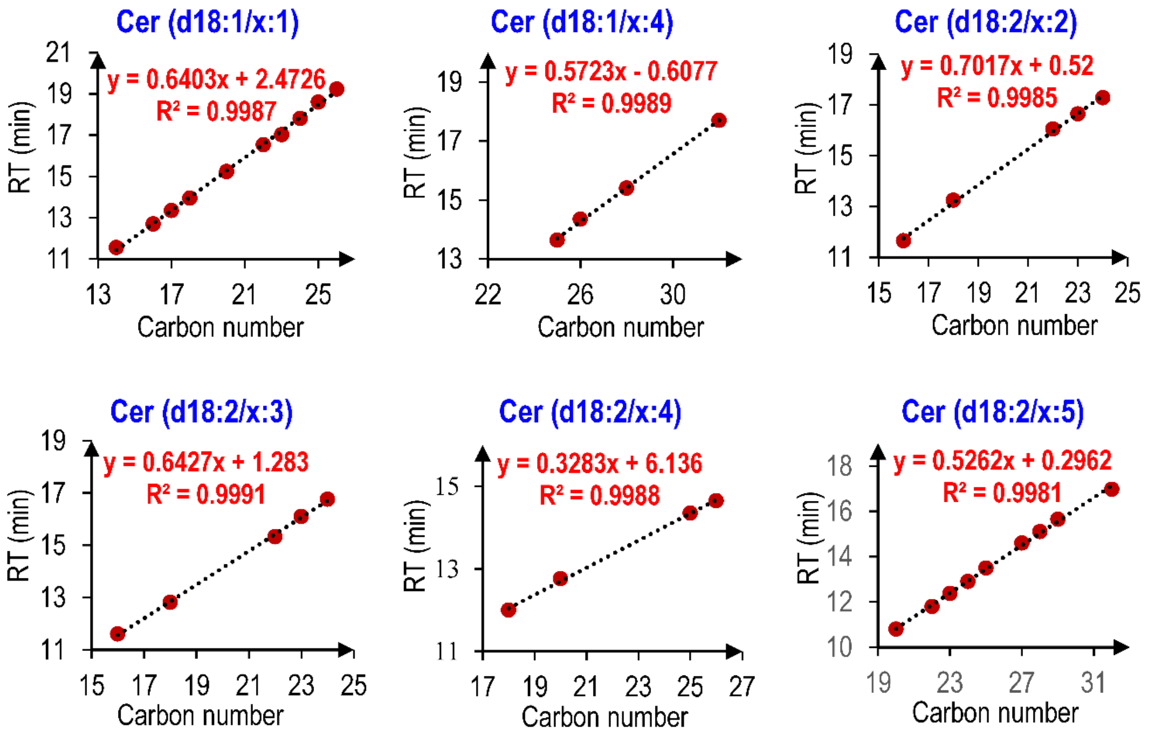

Figure S7. Effects of FTY720 and sphingoid base and ceramide fractions on the proliferation inhibition ratio in LPS-induced mouse splenic lymphocytes (A) and Con A-induced mouse splenic lymphocytes (B). Data were presented as the mean ± SD of five independent experiments.

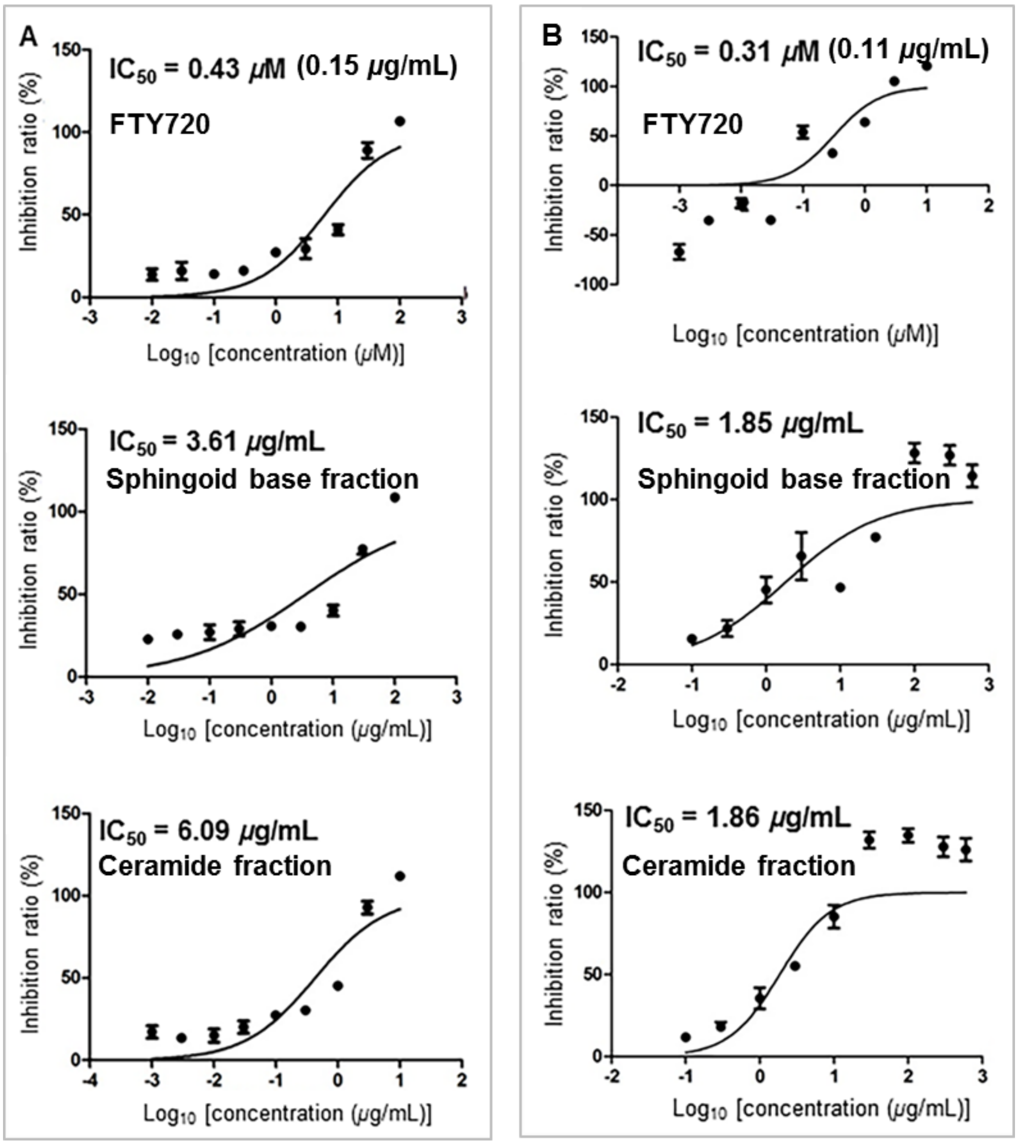

Supplement: Supplementary Information [file srep38641-s1.pdf]
